# A Comparison of Methods for Gene-Based Testing That Account for Linkage Disequilibrium

**DOI:** 10.3389/fgene.2022.867724

**Published:** 2022-05-05

**Authors:** Ozan Cinar, Wolfgang Viechtbauer

**Affiliations:** Department of Psychiatry and Neuropsychology, Maastricht University, Maastricht, Netherlands

**Keywords:** genome-wide association studies, gene-based testing, combining p-values, correlated tests, linkage disequilibrium

## Abstract

Controlling the type I error rate while retaining sufficient power is a major concern in genome-wide association studies, which nowadays often examine more than a million single-nucleotide polymorphisms (SNPs) simultaneously. Methods such as the Bonferroni correction can lead to a considerable decrease in power due to the large number of tests conducted. Shifting the focus to higher functional structures (e.g., genes) can reduce the loss of power. This can be accomplished via the combination of *p*-values of SNPs that belong to the same structural unit to test their joint null hypothesis. However, standard methods for this purpose (e.g., Fisher’s method) do not account for the dependence among the tests due to linkage disequilibrium (LD). In this paper, we review various adjustments to methods for combining *p*-values that take LD information explicitly into consideration and evaluate their performance in a simulation study based on data from the HapMap project. The results illustrate the importance of incorporating LD information into the methods for controlling the type I error rate at the desired level. Furthermore, some methods are more successful in controlling the type I error rate than others. Among them, Brown’s method was the most robust technique with respect to the characteristics of the genes and outperformed the Bonferroni method in terms of power in many scenarios. Examining the genetic factors of a phenotype of interest at the gene-rather than SNP-level can provide researchers benefits in terms of the power of the study. While doing so, one should be careful to account for LD in SNPs belonging to the same gene, for which Brown’s method seems the most robust technique.

## 1 Introduction

Genome-wide association (GWA) studies are commonly used to investigate the contribution of genetic variants to the risk of developing certain diseases (Manolio, 2010). In a typical GWA study, large quantities of single-nucleotide polymorphisms (SNPs) are genotyped to examine their association with some phenotype of interest (e.g., the presence or absence of a disease) or their interaction with some environmental factor (Baranzini et al., 2009; Jiao et al., 2015). However, the availability of genotype information for such a large number of SNPs will either lead to a high rate of type I errors or requires stringent corrections for multiple testing, which in turn inflates the number of type II errors (Johnson et al., 2010).

In particular, the probability of falsely rejecting an individual null hypothesis (e.g., that a SNP is unrelated to the outcome) is set a priori to a specific value by the researcher. This *pointwise error rate* (or error rate per hypothesis) is conventionally set to *α*_*p*_ = 0.05. However, the *familywise error rate*, $\alpha_{s}=1-{\left(1-\alpha_{p}\right)}^{k}$ (i.e., the probability of falsely rejecting at least one of *k* true null hypotheses) quickly increases when testing a large number of independent hypotheses. A common method to control the familywise error rate is the Bonferroni correction (Bland and Altman, 1995) that sets the pointwise error rate to *α*_*p*_/*k*, which in turn keeps *α*_*s*_ below the desired type I error rate (Shaffer, 1995). Considering that nowadays around a million SNPs are genotyped in a typical GWA study (Manolio, 2010), the commonly used significance threshold of 5 × 10^–8^ in such studies is loosely based on the Bonferroni correction (Johnson et al., 2010; Huang et al., 2012).

As a consequence of the decreased significance threshold, rejection of a null hypothesis becomes more difficult, whether it be a true null hypothesis or not. Therefore, the Bonferroni correction also increases the type II error rate (i.e., the probability of failing to reject a false null hypothesis), which in turn decreases power. Although other multiple testing correction methods have been developed that lead to less severe reductions in power (Holm, 1979; Simes, 1986; Hochberg, 1988; Hommel, 1988; Benjamini and Hochberg, 1995; Conneely and Boehnke, 2007), the reduction can still be severe due to the large number of SNPs considered in a typical GWA study (Narum, 2006).

A promising approach for mitigating this severe loss of power is to shift the focus of the analyses to higher functional structures such as genes (known as gene-based testing) or sets of genes that belong to common pathways (Lehne et al., 2011). As a result, the number of hypotheses tested declines dramatically (e.g., to 25,000–30,000 when testing at the gene level) and hence power is not as severely impacted when a correction for multiple testing is then applied. Furthermore, by aggregating signals from multiple SNPs, gene-based testing can be more appropriate for understanding the genetic structure of complex diseases (Liu et al., 2010; Chung et al., 2019).

Although the joint contribution of the SNPs in a gene can be examined with multi-locus tests, such as Hotelling’s *T* ^2^ (Chapman and Whittaker, 2008; Moskvina et al., 2012), such approaches require access to the raw genomic data which may not be available. In the absence of the raw data, we can test the joint null hypothesis of the SNPs that belong to a gene by combining their individual *p*-values into an overall *p*-value. A wide variety of methods have been described in the literature for combining independent tests of hypotheses (Pearson, 1938; Lancaster, 1949; Stouffer et al., 1949; Wilkinson, 1951; Lipták, 1958; Becker, 1994). Among these, Fisher’s method (Fisher, 1932) may be the best-known one, which also has high relative efficiency asymptotically when compared to other methods (Littell and Folks, 1971; Littell and Folks, 1973). However, Fisher’s method, like many other methods for combining tests of hypotheses, assumes that the *p*-values are independent of each other. This assumption is known to be violated in the present context as SNPs are often in linkage disequilibrium (LD), that is, the alleles at different loci exhibit non-random associations (Slatkin, 2008). As a consequence, Fisher’s method does not provide nominal results, typically leading to an inflation in the type I error rate (Moskvina et al., 2011).

Several attempts have been made to adjust methods for combining *p*-values such that they take dependence into consideration. Brown (1975) proposed an adjustment to Fisher’s method for combining the results of dependent tests that has been used for gene-based testing (Moskvina et al., 2011; Zhang et al., 2020), whereas several other authors have described the use of principal component analysis (PCA) on the LD correlation matrix to estimate the effective number of tests (Cheverud, 2001; Nyholt, 2004; Li and Ji, 2005; Gao et al., 2008; Galwey, 2009), which in turn can be combined with various multiple testing correction procedures. In addition, several authors have applied permutation tests or other permutation-type procedures to account for the dependence (Lin, 2005; Liu et al., 2010). However, while permutation tests are often considered a ‘gold standard’ approach, such methods are computationally very demanding especially in GWA studies. Furthermore, proper permutation tests require access to the raw data which can be another limitation. A promising way to mimic the results of permutation tests (without needing the raw data and requiring a fraction of the time) is to generate pseudo replicates of the test statistics assuming they follow a multivariate normal distribution under the null hypothesis. These pseudo test statistics are then converted into SNP-level *p*-values which can be used to generate an empirical distribution of the combined *p*-value under the null hypothesis that takes the degree of LD into consideration (Liu et al., 2010; Li et al., 2011).

The statistical properties (i.e., type I error rate and power) of these methods have been examined in previous research (Lin, 2005; Conneely and Boehnke, 2007; Chapman and Whittaker, 2008; Johnson et al., 2010; Moskvina et al., 2011; Wen and Lu, 2011; Alves and Yu, 2014). However, there are still several points that have not been considered so far. First, none of the studies have performed an extensive comparison among all methods on a genome-wide scale simultaneously. Furthermore, PCA-based approaches have only been combined with the Bonferroni correction (or with Tippett’s method; see below), although they can also be used to modify other tests (e.g., Fisher’s method) to account for dependence among the *p*-values. In addition, it is unknown whether the statistical properties of the correlations (e.g., their central tendency or spread) used to quantify the degree of LD might affect the performance of the methods. Moreover, some theoretical properties of the methods have not received sufficient attention. Most importantly, Brown’s generalization of Fisher’s method only applies to one-sided tests (Brown, 1975). This property is especially problematic in GWA studies, since tests of the association between the SNPs and the phenotype of interest are typically two-sided (Laird and Lange, 2010). An extension of Brown’s method to two-sided tests has been described (Yang et al., 2016); however, its performance in the present context has yet to be investigated.

In this article, we review a variety of methods for combining *p*-values that can be used for gene-based testing and describe how LD can be directly incorporated into these methods. While doing so, an important goal is to provide a more complete description of how methods for combining *p*-values and adjustment techniques can be combined. For example, we will describe how an estimate of the effective number of tests can be used to adjust Fisher’s method. Furthermore, we describe how all methods for combining *p*-values can be adjusted with an empirical distribution obtained using a pseudo-permutation approach. We also discuss the generalization of Brown’s method to two-sided tests. Finally, we compare the type I error rate and power of the methods based on a genome-wide Monte Carlo simulation study using LD matrices derived from the International HapMap Project (The International HapMap Consortium, 2003).

## 2 Methods

For a collection of *i* = 1, … , *k* SNPs that belong to a gene (or pathway), let *p*_1_, … , *p*_*k*_ denote the *p*-values obtained when testing the association of each SNP with some phenotype of interest (or the interaction of each SNP with some other variable). We use *H*_0*i*_ to denote the null hypothesis corresponding to the *i*th SNP. Since we are only interested in testing for association regardless of directionality, we assume that the *p*-values are derived from two-sided tests. Moreover, we assume that the tests have nominal properties, so that *p*_*i*_ ∼Uniform (0, 1) when *H*_0*i*_ is true. Depending on the type of test used for deriving the *p*-values, this assumption may only be true asymptotically (i.e., if the sample size underlying the tests is large). For the purposes of describing the methods, we still make this assumption, but return to this issue in the discussion section.

Instead of considering each of the *p*-values and null hypotheses individually, the goal is to combine the information from the individual tests into one that tests the gene as a whole. To be precise, the goal is to test the joint null hypothesis that none of the SNPs in the gene are associated with the phenotype (i.e., *H*_0*i*_ is true for all tests) against the alternative that at least one SNP is associated. We will now describe a variety of methods for this purpose.

### 2.1 The Bonferroni Method

The Bonferroni correction (Bland and Altman, 1995) is a method that was originally developed to control the familywise error rate when conducting multiple hypothesis tests. In order to apply the correction, the threshold for significance is adjusted by dividing the pointwise error rate, *α*_*p*_, by the number of simultaneous tests, *k*. Alternatively, we can adjust the individual *p*-values by multiplying them with *k*. Any test whose adjusted *p*-value is then equal to or less than *α*_*p*_ is declared significant (Simes, 1986).

Although not typically described in this manner, the Bonferroni method can also be used as a method for combining *p*-values. In particular, if any one of the adjusted *p*-values is significant, then the joint null hypothesis is automatically rejected. In the context of GWA studies, this means that if at least one SNP is significantly associated with the phenotype of interest, then the gene that this SNP belongs to is considered significant. Accordingly, the combined *p*-value for a gene is given by

$$p=\min\left(1,\min\left(p_{1},\ldots ,p_{k}\right)\times k\right),$$ (1)

where min(1, … ) simply ensures that the combined *p*-value cannot exceed 1.

Contrary to popular belief, the Bonferroni correction does not make any assumptions about the degree of dependence among the *p*-values (Goeman and Solari, 2014). In other words, regardless of the degree of dependence among the tests from which the *p*-values are derived, the method guarantees that the type I error rate is no larger than the desired nominal rate. This makes the methods particularly interesting for gene-based testing, where we know that the tests are likely to be dependent due to LD.

### 2.2 Methods Assuming Independence

In this subsection, we will describe methods that assume that the tests, and hence the *p*-values to be combined, are independent. Adjustments thereof will be considered later.

#### 2.2.1 Tippett’s Method

Tippett’s method (Tippett, 1931), also known as the Dunn-Šidák correction for multiple testing (Šidák, 1957; Dunn, 1958), follows from the fact that the familywise type I error rate for *k* independent tests, $\alpha_{s}=1-{\left(1-\alpha_{p}\right)}^{k}$ , will equal a desired nominal rate, *α*, if we set *α*_*p*_ = 1 − (1 − *α*)^1/*k*^. Hence, the joint null distribution can be rejected if min(*p*_*i*_) ≤ 1 − (1 − *α*)^1/*k*^. Analogously, we can use

$$p=1-{\left(1-\min\left(p_{1},\ldots ,p_{k}\right)\right)}^{k}$$ (2)

as the *p*-value for the gene. As opposed to the Bonferroni method, which is slightly conservative even when all tests are independent, the method provides exact control of the type I error rate, but only under independence.

#### 2.2.2 Binomial Test

Under the joint null hypothesis, *r* ∼Binomial (*k*, *α*_*p*_) where *r* denotes the number of tests that are significant at *α*_*p*_. Therefore, we can reject the joint null hypothesis if

$$p=\overset{k}{\underset{x=r}{\sum }}\left(\begin{array}{c} k\\ x \end{array}\right)\alpha_{p}^{x}{\left(1-\alpha_{p}\right)}^{k-x}$$ (3)

is equal to or less than the desired type I error rate (Wilkinson, 1951). Intuitively, we can interpret this method as a test of “excess significance” of the SNPs within a gene. For example, the chances of finding *r* = 10 or more significant SNPs at *α*_*p*_ = 0.05 in a gene with 100 independent SNPs is approximately *p* = 0.028, which would be significant at *α* = 0.05.

#### 2.2.3 Fisher’s Method

Assuming that *p*_*i*_ ∼Uniform (0, 1) under the null hypothesis of no association, it is easy to show that −2 ln(*p*_*i*_) is chi-square distributed with 2 degrees of freedom. Hence, the combined test statistic

$$X^{2}=-2\overset{k}{\underset{i=1}{\sum }}\ln\left(p_{i}\right)$$ (4)

follows a chi-squared distribution with 2*k* degrees of freedom under the joint null hypothesis (Fisher, 1932). The *p*-value for the gene can therefore be computed with *p* = 1 − *F* (*X* ^2^, 2*k*), where *F* (⋅, 2*k*) denotes the cumulative distribution function of a chi-square distribution with 2*k* degrees of freedom.

#### 2.2.4 Stouffer’s Method

Let Φ(⋅) denote the cumulative distribution function of the standard normal distribution and Φ^−1^(⋅) its inverse. Since Φ^−1^ (1 − *p*_*i*_) follows a standard normal distribution under *H*_0*i*_, $z=\sum_{i=1}^{k}\Phi^{-1}\left(1-p_{i}\right)/\sqrt{k}∼\text{Normal}\left(0,1\right)$ under the joint null hypothesis (Stouffer et al., 1949). The *p*-value for a gene is then computed with *p* = 1 − Φ(*z*).

### 2.3 Incorporating Linkage Disequilibrium

Except for the Bonferroni method, all methods described in the previous section assume that the tests are independent. Therefore, under this assumption (and the assumptions stated at the beginning of this section), these methods are guaranteed to have a type I error rate equal to the desired *α* level (for the binomial test, the type I error rate is ≤α due to the discrete nature of the binomial distribution). On the other hand, when the independence assumption is violated, the true type I error rate may deviate from *α* in either direction, but usually leading to inflation (i.e., the joint null is rejected too often). In comparison, the Bonferroni method makes no assumptions about the degree of dependence among the tests and is guaranteed to have a rejection rate that is no larger than *α*, but can be quite conservative under dependence. We will therefore now consider adjustments to the methods that account for dependence among the tests and that can bring their type I error rate closer to *α*.

#### 2.3.1 Effective Number of Tests

One potential approach to adjust the previous methods is to quantify the degree of dependence between the tests, estimate the effective number of independent tests based on this information, and incorporate this estimate into the methods described above.

The degree of dependence between the tests is closely related to the strength of the association between the SNPs. The latter can be quantified with various statistics (e.g., *D*, *D*′, *r*, or *r* ^2^) expressing the degree of LD between pairs of SNPs (Laird and Lange, 2010). We can use one of these measures to construct a *k* × *k* association matrix for all SNPs, sometimes called an “LD map”. The effective number of tests can then be estimated based on this association matrix. A variety of approaches have been described in the literature for this purpose (Cheverud, 2001; Nyholt, 2004; Li and Ji, 2005; Gao et al., 2008; Galwey, 2009). A common feature of all methods is that they start by applying PCA to the association matrix. We use *λ*_*i*_ to denote the *i*th eigenvalue extracted from the PCA.

The method proposed by Cheverud (2001) and Nyholt (2004) estimates the effective number of tests with

$$k_{\text{eff}}^{\text{CN}}=1+\left(k-1\right)\left(1-\frac{\mathrm{Var}\left(\lambda \right)}{k}\right),$$ (5)

where Var(*λ*) is the variance of the *k* eigenvalues. On the other hand, Li and Ji (2005) suggested the formula

$$k_{\text{eff}}^{\text{LJ}}=\overset{k}{\underset{i=1}{\sum }}f\left(\left|\lambda_{i}\right|\right),$$ (6)

where

$$f\left(x\right)=I\left(x\geq 1\right)+\left(x-\left\lfloor x\right\rfloor \right)$$ (7)

and ⌊⋅⌋ is the floor function. According to the method by Gao et al. (2008), we first sort the eigenvalues in decreasing order, letting *λ*_(1)_ denote the largest and *λ*_(*k*)_ the smallest eigenvalue. Then the effective number of tests is defined as

$$k_{\text{eff}}^{\text{GAO}}=\min\left(x\right)\;\text{such that}\;\frac{\sum_{i=1}^{x}\lambda_{\left(i\right)}}{\sum_{i=1}^{k}\lambda_{\left(i\right)}}>C,$$ (8)

where *C* is a user-defined parameter and usually chosen to be 0.995. Finally, Galwey (2009) proposed to estimate the effective number of tests with

$$k_{\text{eff}}^{\text{GAL}}=\frac{{\left(\sum_{i=1}^{k}\sqrt{\lambda_{i}^{\prime }}\right)}^{2}}{\sum_{i=1}^{k}\lambda_{i}^{\prime }},$$ (9)

where $\lambda_{i}^{\prime }=\max\left(0,\lambda_{i}\right)$ .

All of the methods described above have the following desirable properties. When applied to an identity matrix (i.e., when there is no association between any pair of SNPs), then *k*_eff_ = *k*, so that the effective number of tests is equal to the number of SNPs. An exception to this property can occur with $k_{\text{eff}}^{\text{GAO}}$ . Depending on the value of *C* and the number of tests, it can happen that the effective number of tests is then estimated to be less than *k* (i.e., when *k* (1 − *C*) > 1 then $k_{\text{eff}}^{GAO}<k$ ). On the other hand, when all of the SNPs are perfectly associated (i.e., the correlation matrix is equal to a *k* × *k* matrix of 1’s), then *k*_eff_ = 1. In essence, the same test is then repeated *k* times, yielding identical results, so that effectively only a single test has been carried out. However, the methods differ in how association matrices that fall in between these two extremes are handled, yielding varying estimates of the effective number of tests between 1 and *k*.

Once *k*_eff_ has been estimated with one of these approaches, it can be used to adjust each of the methods for combining *p*-values described earlier. For the Bonferroni and Tippett’s methods, we substitute *k*_eff_ for *k* so that

$$p=\min\left(1,\min\left(p_{1},\ldots ,p_{k}\right)\times k_{\text{eff}}\right).$$ (10)

and

$$p=1-{\left(1-\min\left(p_{1},\ldots ,p_{k}\right)\right)}^{k_{\text{eff}}}$$ (11)

are then the *p*-values for the gene. For the binomial test, we first define $\tilde{r}=\left\lfloor r\times \frac{k_{\text{eff}}}{k}\right\rfloor$ as the adjusted (i.e., effective) number of significant SNPs within the gene. Then the *p*-value for the gene is computed with

$$p=\overset{k_{\text{eff}}}{\underset{x=\tilde{r}}{\sum }}\left(\begin{array}{c} k_{\text{eff}}\\ x \end{array}\right)\alpha_{p}^{x}{\left(1-\alpha_{p}\right)}^{k_{\text{eff}}-x}.$$ (12)

Use of the floor function for computing $\tilde{r}$ may be conservative, but we consider this preferable over rounding and the risk of a too liberal test. Fisher’s method can be adjusted by replacing the degrees of freedom of the chi-square distribution with 2*k*_eff_ and adjusting the test statistic with ${\tilde{X}}^{2}=\frac{k_{\text{eff}}}{k}\times X^{2}$ . Hence, the *p*-value for the gene is then computed with $p=1-F\left({\tilde{X}}^{2},2k_{\text{eff}}\right)$ . Finally, for Stouffer’s method, we let $\tilde{z}=\sqrt{\frac{k_{\text{eff}}}{k}}\times z$ denote the adjusted test statistic and hence $p=1-\Phi \left(\tilde{z}\right)$ is then the *p*-value for the gene.

#### 2.3.2 Methods Based on Empirically-Derived Null Distributions

Another approach to account for dependence is to make use of permutation testing (Johnson et al., 2010; Moskvina et al., 2011). The idea is to empirically derive the null distribution of the test statistic of interest by reshuffling the data in such a way that relevant features of the data structure are preserved except for the actual association being tested. For example, when testing for the association between each SNP and case-control status, reshuffling the status variable breaks any existing associations, but keeps the LD structure of the SNPs intact. Hence, any dependence among the *p*-values to be combined using one of the methods described earlier is automatically incorporated into the null distribution. The *p*-value for a gene is then computed from the percentile of the actually observed test statistic under the empirical null distribution. Note that in the present case, the test statistic of interest is actually a *p*-value itself, so letting *p*_*j*_ denote the combined *p*-value based on the *j*th permutation of the data (with *j* = 1, … , *s*) and *p*_obs_ the observed combined *p*-value, the *p*-value for a gene is then given by $p=\sum_{j=1}^{s}I\left(p_{j}\leq p_{\text{obs}}\right)/s$ .

Permuting the data in the manner described above requires access to the raw data, so that the phenotype variable can be reshuffled and the test of association can be conducted for each SNP. In addition, repeatedly computing the test of association for each SNP within a gene can be computationally demanding. We can reduce the computational burden and eliminate the dependence upon the raw data by directly generating *p*-values based on an association matrix that reflects the degree of LD among the SNPs (Liu et al., 2010) which may be obtained from a reference population and not necessarily the given data.

In particular, let *R* denote the LD association matrix constructed from the correlations among the SNPs. We can then quickly generate a large number (*s*) of samples from a multivariate normal distribution with a true mean vector equal to zeros and covariance matrix *R*. Let *Z* denote the *s* × *k* matrix of these values and *P* = 2 (1 − Φ(|*Z*|)) the matrix of two-sided *p*-values obtained by applying Φ(⋅) element-wise. For each row in *P*, we then apply one of the methods for combining *p*-values, yielding *p*_*j*_. The *p*-value for a gene is then again computed as described above.

#### 2.3.3 Methods Derived Under Dependence

The last set of methods we will consider are modifications of Fisher’s and Stouffer’s method so that dependence among the tests is directly taken into consideration.

##### 2.3.3.1 Brown’s Method

The first adjustment is based on Brown (1975) who proposed a modification of Fisher’s method for combining the results of correlated one-sided *z*-tests. If the *p*-values are not independent, *X* ^2^ has expected value E(*X* ^2^) = 2*k* and variance $\mathrm{Var}\left(X^{2}\right)=4k+2\sum_{i=1}^{k-1}\sum_{j>i}^{k}\text{Cov}\left(-2\ln\left(p_{i}\right),-2\ln\left(p_{j}\right)\right)$ , where the covariance between two −2 ln (⋅)-transformed *p*-values is given by

$$\text{Cov}\left(-2\ln\left(p_{i}\right),-2\ln\left(p_{j}\right)\right)=4\overset{+\infty }{\underset{-\infty }{\iint }}\ln\left(1-\Phi \left(z_{i}\right)\right)\times \ln\left(1-\Phi \left(z_{j}\right)\right)f\left(z_{i},z_{j}\right)dz_{i}dz_{j}-4,$$ (13)

where (*z*_*i*_, *z*_*j*_) is assumed to follow a bivariate standard normal distribution with correlation equal to the correlation among the two SNPs and *f*(*z*_*i*_, *z*_*j*_) denotes the joint probability density function of this distribution. The covariance term can be computed using numerical integration, although Brown (1975) also proposed a closed-form approximation that avoids this step. Next, we assume that *X* ^2^ follows a scaled chi-squared distribution, i.e., $X^{2}∼c\chi_{f}^{2}$ (or equivalently, $X^{2}/c∼\chi_{f}^{2}$ ), where $\chi_{f}^{2}$ denotes a chi-squared distributed random variable with *f* degrees of freedom, and then approximate this distribution by equating its first two moments to the expected value and variance of *X* ^2^ as calculated above. That is, for $X^{2}∼c\chi_{f}^{2}$ , it follows that E (*X* ^2^) = *cf* and Var(*X* ^2^) = 2*c* ^2^ *f*, which implies $f=2{\left(\text{E}\left(X^{2}\right)\right)}^{2}/\mathrm{Var}\left(X^{2}\right)$ and *c* = Var(*X* ^2^)/2E (*X* ^2^). The *p*-value for a gene is then computed with *p* = 1 − *F*(*X* ^2^/*c*, *f*), where *F*(⋅, *f*) denotes the cumulative distribution function of a chi-square distribution with *f* degrees of freedom.

As given above, the method is only applicable to one-sided tests. However, in GWA studies, the association between the phenotype and the SNPs is typically examined with two-sided tests. We can easily extend Brown’s method to two-sided tests by computing the covariance with

$$\text{Cov}\left(-2\ln\left(p_{i}\right),-2\ln\left(p_{j}\right)\right)=4\overset{+\infty }{\underset{-\infty }{\iint }}\ln\left(2\left(1-\Phi \left(\left|z_{i}\right|\right)\right)\right)\times \ln\left(2\left(1-\Phi \left(1-|z_{j}|\right)\right)\right)f\left(z_{i},z_{j}\right)dz_{i}dz_{j}-4,$$ (14)

with (*z*_*i*_, *z*_*j*_) and *f* (*z*_*i*_, *z*_*j*_) as defined above (Yang et al., 2016). The remaining steps of the method are unchanged.

##### 2.3.3.2 Strube’s Method

Finally, Stouffer’s method can also be generalized to consider the dependence among tests (Strube, 1985). To do so, we assume (as in Brown’s method) that the test statistics that generated the *p*-values follow a multivariate normal distribution where the correlations among the test statistics are given by the correlations among the SNPs. We then compute

$$z=\frac{\sum_{i=1}^{k}\Phi^{-1}\left(1-p_{i}\right)}{\sqrt{\mathrm{Var}\left(\sum_{i=1}^{k}\Phi^{-1}\left(1-p_{i}\right)\right)}},$$ (15)

where $\mathrm{Var}\left(\sum_{i=1}^{k}\Phi^{-1}\left(1-p_{i}\right)\right)=k+2\sum_{i=1}^{k-1}\sum_{j>i}^{k}\text{Cov}\left.\left(\Phi^{-1}\left(1-p_{i}\right),\Phi^{-1}\left(1-p_{j}\right)\right)\right)$ . The challenge is again the computation of the covariance term, which in this case is given by

$$\text{Cov}\left(\Phi^{-1}\left(1-p_{i}\right),\Phi^{-1}\left(1-p_{j}\right)\right)=\overset{+\infty }{\underset{-\infty }{\iint }}\Phi^{-1}\left(1-2\left(1-\Phi \left(\left|z_{i}\right|\right)\right)\right)\times \Phi^{-1}\left(1-2\left(1-\Phi \left(\left|z_{j}\right|\right)\right)\right)f\left(z_{i},z_{j}\right)dz_{i}dz_{j}$$ (16)

and which can again be computed using numerical integration. Then the combined *p*-value is calculated with *p* = 1 − Φ(*z*).

### 2.4 Illustrative Example

The methods for combining *p*-values described in the previous section can yield conflicting conclusions. In particular, while the (unadjusted) Bonferroni method controls the type I error rate even under dependence, it may fail to detect significant associations when combining non-independent *p*-values due to its conservative behaviour in such contexts. We present an example to illustrate this point.

Assche et al. (2017) reported the results of a candidate gene study based on a sample of 982 Caucasian adolescents, analyzing 4,947 SNPs clustered in 263 genes known to be involved in neurotransmission. The outcome of interest was the (log-transformed) score on the Center for Epidemiologic Studies Depression Scale (Radloff, 1977). The association between each SNP and the outcome was tested using an additive model (Laird and Lange, 2010). The resulting *p*-values were then combined within each gene using Brown’s method. The results showed that a small number of genes were significantly associated with the phenotype at *α* = 0.05.

For illustration purposes, we obtained the combined *p*-values for two genes (*GRID2IP* and *ARNTL2*) with all of the methods described above. LD maps were calculated using the LD() function of the genetics package in R (Warnes et al., 2013), using the allelic correlation to measure the degree of association between the SNPs within each gene. The combined *p*-values were then obtained using the poolr package in R (Cinar and Viechtbauer, 2020). Empirical null distributions were generated as described earlier using *s* = 10^6^ samples.

### 2.5 Simulation Study

To compare the performance of the various methods more systematically, we conducted a simulation study based on HapMap phase II + III data (The International HapMap Consortium, 2003) so that the results are representative of real genotype and LD information across the whole genome. Since genetic recombination breaks down disequilibria among the SNPs over time, LD tends to be weaker in older populations (Koch et al., 2013). We therefore used information from the TSI (Italian) sample from a somewhat younger population to avoid LD maps overwhelmed with negligible pairwise LD values. The sample contained *n* = 102 individuals and 1,421,526 SNPs with their chromosome and position information.

We focus on autosomal chromosomes and excluded the sex chromosomes. Furthermore, insertions and deletions (INDELs) in the data (Mills et al., 2006) were removed. SNPs were assigned to genes using the biomaRt package in R through the Ensembl database (Hubbard et al., 2002; Durinck et al., 2005, 2009). SNPs that were not assigned to a gene were excluded while SNPs that were assigned to multiple genes (due to overlapping genes) were kept in the study. After the assignment of SNPs to genes, the data included 915,259 SNPs in 30,910 genes. The number of SNPs per gene ranged from 2 to 3,178 with a mean (SD) of 29.61 (68.68) and a median of 12. Missing genotypes were then imputed using the MaCH software (Li et al., 2010). Next, LD maps (again using allelic correlations) were computed as described above. For LD maps that were not positive definite, the nearest positive definite correlation matrices were obtained with the nearPD() function of the Matrix package (Bates and Maechler, 2015). Finally, genotypes were coded in an additive manner (i.e., 0/1/2 coding), corresponding to the number of minor alleles at a locus (Moskvina et al., 2011).

For each gene, we examined the type I error rate of the methods by 1) simulating a dichotomous phenotype variable (e.g., case-control status) for the *n* = 102 individuals based on a Bernoulli distribution with *π* = 0.50, 2) testing the association between each SNP within the gene and the phenotype variable using the Cochran-Armitage trend test (Cochran, 1954; Armitage, 1955), 3) combining the resulting *p*-values with each method described earlier, 4) repeating this process 1,000 times, and 5) calculating the proportion of times that the gene is declared significant at *α* = 0.05 according to each method. For methods that make use of empirical distributions, we generated these distributions as described earlier using *s* = 10^5^ samples. Since the Cochran-Armitage trend test does not require that the Hardy-Weinberg equilibrium (HWE) assumption holds (Laird and Lange, 2010), we did not filter out SNPs that violate this assumption. Also, we note here that our goal was to examine the performance of the various methods based on individual genes, not at a whole genome-wide level. Therefore, we tested each gene at *α* = 0.05, not at some level corrected for multiple testing. However, if a particular methods controls the type I error rate on individual genes, then it will also do so when testing all genes at *α* = 0.05/*g*, where *g* denotes the total number of genes tested.

To examine the power of the methods, the same steps as described above were repeated, but the probability of having case status was now made a logistic function of one or multiple SNPs within the gene, that is, for the *j*th individual in the data set, we set the probability of having case status equal to

$$\pi_{j}=\frac{\exp\left(\beta_{0}+\sum_{i=1}^{k}x_{ij}\beta_{i}\right)}{1+\exp\left(\beta_{0}+\sum_{i=1}^{k}x_{ij}\beta_{i}\right)},$$ (17)

where *x*_*ij*_ denotes the number of minor alleles for the *i*th SNP of the *j*th individual, *β*_*i*_ determines how strongly the *i*th SNP is related to case-control status, and

$$\beta_{0}=-\frac{\sum_{j=1}^{n}\sum_{i=1}^{k}x_{ij}\beta_{i}}{n},$$ (18)

so that approximately half of the *n* individuals were assigned to case status and the other half were controls.

This part of the simulation involved several scenarios with different features. In the first set of conditions, a single SNP within a given gene was chosen in each iteration and the corresponding *β*_*i*_ value was set to either 0.2 or 0.5 (two different conditions). In the remaining conditions, we either allowed 5% or 20% of the SNPs within each gene to be associated with the phenotype. We again examined two different effect sizes (0.2 or 0.5) and either set *β*_*i*_ to the effect size value for each selected SNP (non-distributed effect) or distributed the effect over all selected SNPs (e.g., *β*_*i*_ = 0.2/10 for an effect size of 0.2 and 10 selected SNPs). Finally, the selected SNPs were either positioned on a compact region of the gene (for this, a single SNP was randomly chosen among the first *k* × (1 − 0.05) or *k* × (1 − 0.20) SNPs and all consecutive SNPs were then also selected) or were dispersed throughout the gene (for this, significant SNPs were equally spaced throughout the gene). Hence, in addition to the first two conditions where a single SNP was associated with the phenotype with either an effect size of 0.2 or 0.5, we examined another 16 conditions, as all factors (5 vs. 20% of SNPs selected, effect size of 0.2 vs. 0.5, non-distributed vs. distributed effect, non-compact vs. compact SNP selection) were fully crossed.

To examine how sample size impacts the type I error rate and power of the methods, the same steps were repeated but in each iteration, a bootstrap sample of size 102, 500, or 1,000 was generated from the original data (together with the non-bootstrap conditions, we therefore examined 4 different sample size conditions). We included a condition with a bootstrap sample size of 102 to examine whether the performance of the methods differed whether the original data or a bootstrap sample of the same size was used. In total, we therefore examined a total of (1 _type I error_ + 2 _Single SNP_ + 16 _Multiple SNPs_) × 4 _Sample Size_ = 76 different conditions.

The simulation was carried out using R (R Core Team, 2020) and was run on a cluster computer, making use of 144 cores (12 Intel Xeon E5-2,650 2.20 GHz CPUs with 12 cores each) using parallel/multicore processing. Total computation time for the simulation was approximately 20,000 core hours.

## 3 Results

### 3.1 Illustrative Example

For the illustrative example, Table 1 presents the combined *p*-values for the two genes. Heat maps corresponding to the LD structure for these two genes (and the individual *p*-values for the SNPs) are provided in Supplementary Figure S9 as part of the supplementary materials. The table shows that the Bonferroni method fails to detect a significant association between both genes and the phenotype, whereas other approaches (including Brown’s method) suggest a significant association. Interestingly, adjusting the Bonferroni method with two of the PCA-based methods (i.e., $k_{\text{eff}}^{\text{LJ}}$ and $k_{\text{eff}}^{\text{GAL}}$ ) leads to a significant finding at least for *GRID2IP*.

**TABLE 1:** Combined *p*-values for the *GRID2IP* and *ARNTL2* genes based on the methods presented in Section 2 (combined *p*-values that show *non-significant* associations are accentuated in *italic*).

*GRID2IP*	Unadjusted	Cheverud-Nyholt	Li and ji	Gao	Galwey	Empirically derived	Under dependence
*k* = 23		$k_{\text{eff}}^{\text{CN}}=20$	$k_{\text{eff}}^{\text{LJ}}=15$	$k_{\text{eff}}^{\text{GAO}}=18$	$k_{\text{eff}}^{\text{GAL}}=13$		
Bonferroni	*0.068*	*0.060*	0.045	*0.054*	0.039	*0.052*	
Tippett	*0.066*	*0.058*	0.044	*0.052*	0.038	*0.051*	
Binomial	<0.001	<0.001	<0.001	<0.001	<0.001	<0.001	
Fisher	<0.001	<0.001	<0.001	<0.001	<0.001	0.002	0.001
Stouffer	<0.001	<0.001	<0.001	<0.001	<0.001	0.002	<0.001

***ARNTL2***	**Unadjusted**	**Cheverud-Nyholt**	**Li and Ji**	**Gao**	**Galwey**	**Empirically Derived**	**Under Dependence**
***k* = 24**		$k_{eff}^{CN}=22$	$k_{eff}^{LJ}=14$	$k_{eff}^{GAO}=18$	$k_{eff}^{GAL}=14$		
Bonferroni	*0.112*	*0.103*	*0.065*	*0.084*	*0.065*	*0.082*	
Tippett	*0.106*	*0.098*	*0.063*	*0.081*	*0.063*	*0.082*	
Binomial	<0.001	0.001	0.004	0.002	0.004	0.011	
Fisher	<0.001	<0.001	0.003	0.001	0.003	0.020	0.016
Stouffer	0.001	0.001	0.009	0.003	0.009	0.030	0.023

The number of SNPs in the genes are denoted by *k*, whereas $k_{\text{eff}}^{\text{CN}}$ , $k_{\text{eff}}^{\text{LJ}}$ , $k_{\text{eff}}^{\text{GAO}}$ , and $k_{\text{eff}}^{\text{GAL}}$ denote the effective number of tests estimated by the methods specified in the column header.

While this example demonstrates that conclusions can differ depending on the method used, we do not know which of the conclusions drawn above are correct. In other words, the (non-significant) results of the Bonferroni method may be Type II errors (which are then avoided by using other methods) or they may be true negatives (with other methods then leading to Type I errors). The results of the simulation study will provide further insights into the performance of the methods when the true status of each gene is known.

### 3.2 Simulation Study

#### 3.2.1 Type I Error Rates

**FIGURE 1:**
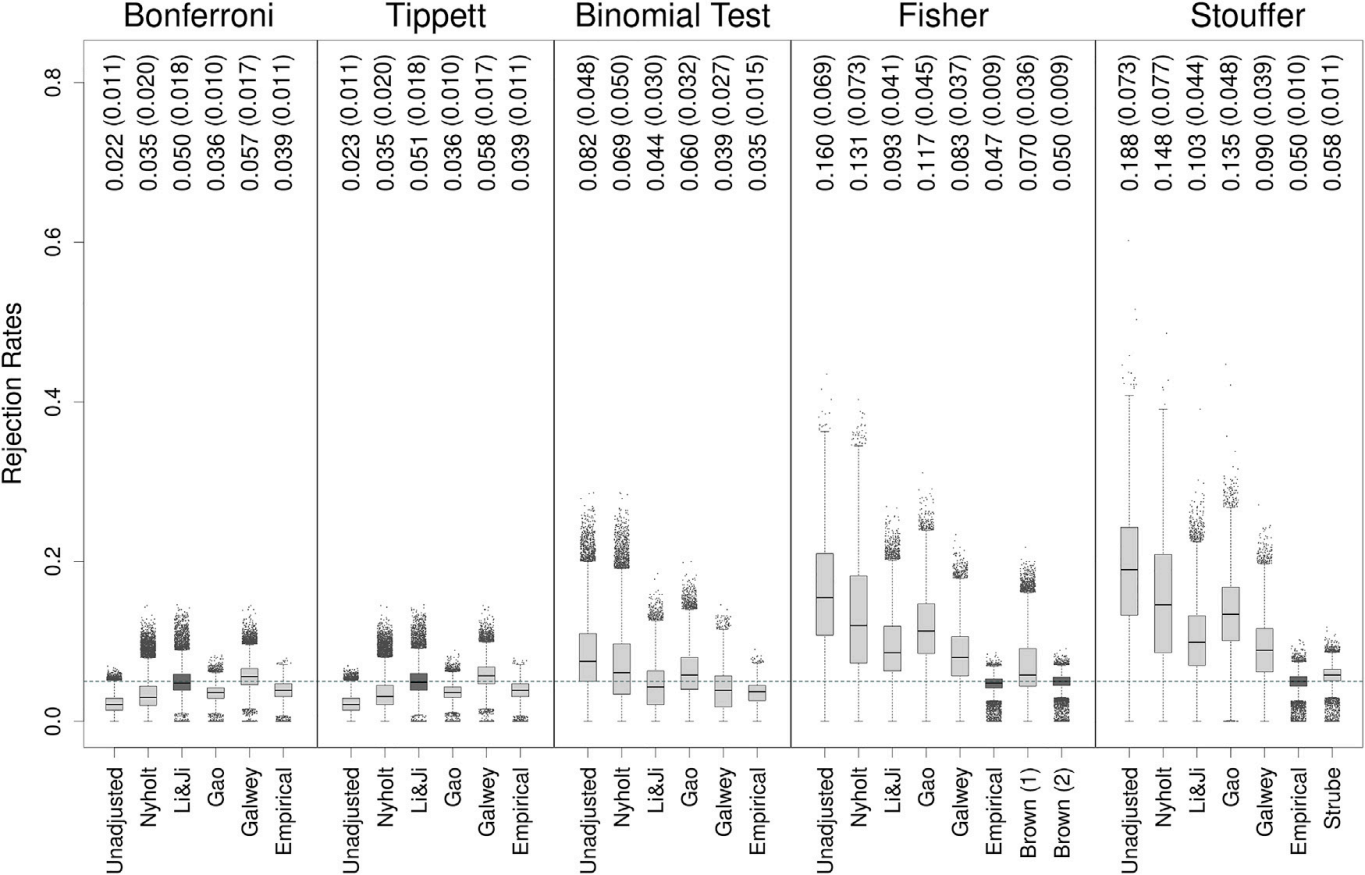
Type I error rates of methods for gene-based testing when applied to the original HapMap data. The numbers above the boxplots show the mean (SD) rejection rates of the methods. The horizontal grey dashed line corresponds to the nominal rejection rate of *α* = 0.05. Brown (1) and Brown (2) refer to the one- and two-sided versions of Brown’s method (i.e., Eqs 13,14, respectively). Individual genes are indicated as points when their rejection rate was more than 1.5 times the interquartile range above the third or below the first quartile.

Figure 1 shows boxplots of the type I error rates of all methods observed on all 30,910 genes applied to the original HapMap dataset (i.e., based on the non-bootstrapped data). Individual genes are indicated as points when their rate was more than 1.5 times the interquartile range above the third or below the first quartile. The mean rejection rates (and SDs) are also indicated in the figure.

None of the unadjusted methods could achieve on average a nominal rejection rate of *α* = 0.05. As expected, the Bonferroni method tended to be conservative, as was Tippett’s method, which produced very similar results to the former method throughout the entire simulation study and which will therefore not be further discussed (note that rates above 0.05 for the Bonferroni method–which occurred for about 1.5% of the genes–reflect simulation error, since we know that the method guarantees that the type I error rate is equal to or less than *α* regardless of the degree of dependence). On the other hand, the remaining methods were generally liberal, at times dramatically so, with the binomial test at least providing an average rejection rate closest to the nominal level.

The adjustments for addressing the dependence did bring the rejection rates closer to the nominal level with varying degrees of success. In fact, when adjusted with the Li & Ji method, the Bonferroni method had a nominal average rejection rate, although this came at the cost of increased variability in the type I error rates, and the occurrence of rates well above the nominal level for particular genes. For the binomial test, the average rates fluctuated around the nominal level, being slightly conservative with the Li & Ji and Galwey adjustments and slightly liberal with the Nyholt and Gao adjustments. In contrast, none of the PCA-based adjustments could bring the average type I error rates of the Fisher and Stouffer methods sufficiently close to *α* = 0.05.

Using empirically-derived null distributions produced rejection rates that were on average reasonably close to the nominal level, especially for Fisher’s and Stouffer’s methods. Moreover, the type I error rates of individual genes had much lower variability than the rates obtained with the PCA-based adjustments. This was also true for the Bonferroni method and the binomial test, but these methods were slightly conservative on average when adjusted in this manner.

The (two-sided) generalization of Fisher’s method to dependent tests (i.e., Brown’s method) yielded a nominal rejection rate on average. Furthermore, the variability (i.e., SD) of the rates for individual genes was lowest compared to all other methods. Quite importantly (mis)application of the one-sided version of Brown’s method (since the *p*-values for the SNPs were computed from two-sided tests) resulted in worse performance (further references to Brown’s method will therefore pertain to the two-sided version unless otherwise stated). On the other hand, the generalization of Stouffer’s method to dependent tests (i.e., Strube’s method) performed reasonably well, although its type I error rate was on average slightly inflated.

Supplementary Figures S1–S3 show the type I error rates of the methods based on bootstrap samples of sizes 102, 500, and 1,000, respectively. A comparison of Figure 1; Supplementary Figure S1 (both with *n* = 102) shows that the performance of the methods was similar regardless of whether they were applied to the original data or to bootstrap samples of the same size. The only exception to this was Stouffer’s method, which became slightly more conservative for the bootstrapped data. Also, the patterns in the type I error rates of the methods were not fundamentally altered when applied to larger sample sizes. Using empirical distributions in combination with Fisher’s and Stouffer’s methods and Brown’s method generally resulted in adequate control of the type I error rate on average and comparatively low variability in the rates for individual genes.

To examine whether the performance of the methods was affected by certain characteristics of the genes, we examined their type I error rates as a function of the (log transformed) number of SNPs in the genes, the average correlation in the LD maps, the degree of variability (i.e., standard deviation) of the correlations, the square-root of the mean squared correlations (SRMSC), the average minor allele frequencies (MAF) of the SNPs in the genes, and the standard deviation (SD) of the MAFs. The SRMSC was of particular interest as it distinguishes genes whose SNPs are independent (SRMSC equal to 0) from genes with SNPs in strong LD regardless of the directionality of the association (SRMSC close to 1). We used locally estimated scatterplot smoothing to visualize the relationship between these characteristics and the rejection rates for each method.

**FIGURE 2:**
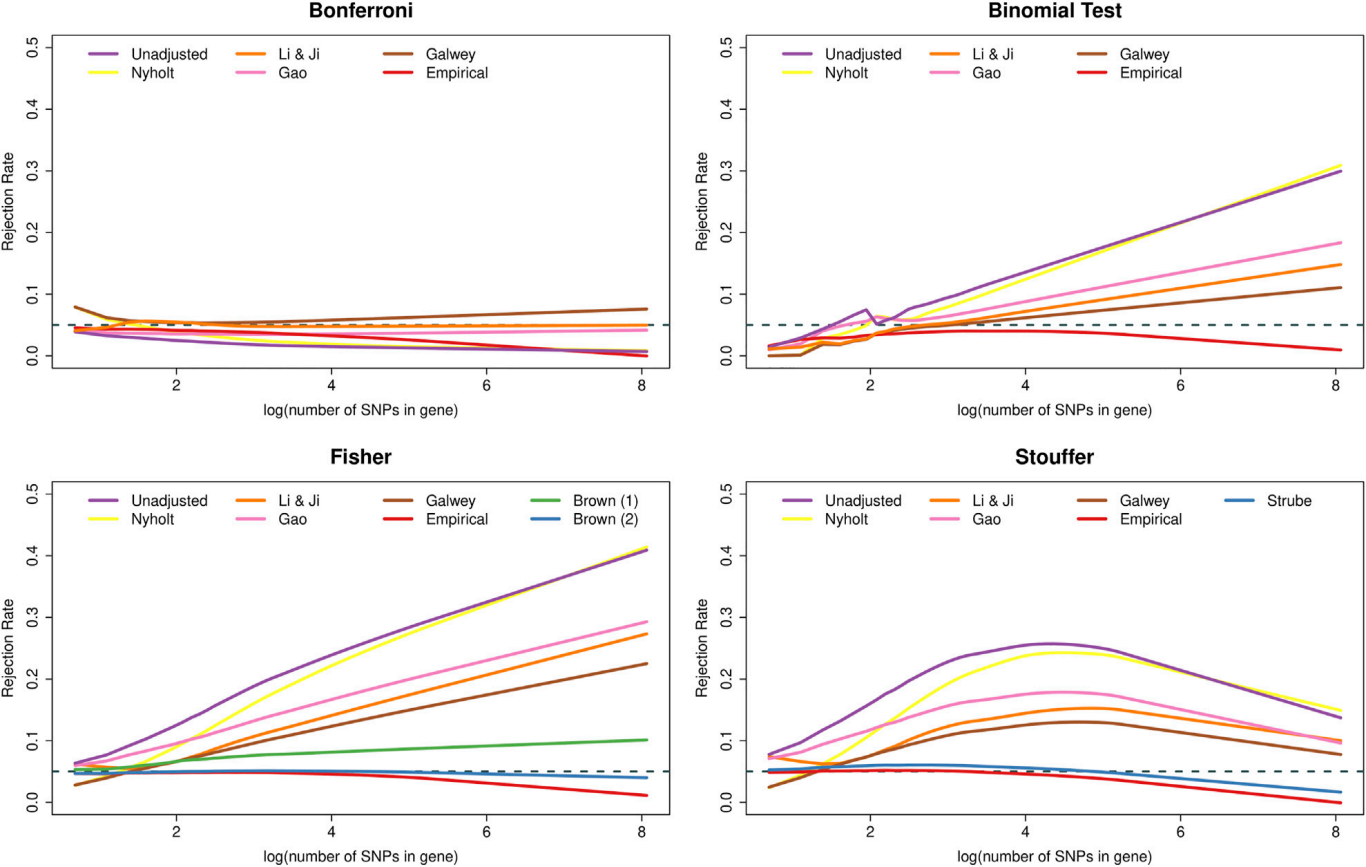
Type I error rates of methods for gene-based testing as a function of the number of SNPs in the genes (log-transformed). The horizontal grey dashed line corresponds to the nominal rejection rate of *α* = 0.05. Brown (1) and Brown (2) refer to the one- and two-sided versions of Brown’s method (i.e., Eqs 13,14, respectively).

Figure 2 shows that the type I error rate of many methods was affected by the number of SNPs in the genes. In particular, the Bonferroni method became increasingly conservative as the number of SNPs increased. Interestingly, this dependence on *k* was essentially removed when using the adjustment of Li & Ji and, to a slightly lesser extent, the adjustment of Gao. In contrast, the binomial test and Fisher’s method became increasingly liberal as a function of *k*, whereas Stouffer’s method displayed non-monotonic behaviour. The PCA-based adjustments helped to reduce the inflation in the type I error rates of these methods, but could not eliminate the dependence on *k*. Furthermore, all methods adjusted based on empirical distributions became increasingly conservative as the number of SNPs increased. Finally, Brown’s method yielded essentially nominal rates regardless of *k*, except for very large genes, where the method became slightly conservative.

**FIGURE 3:**
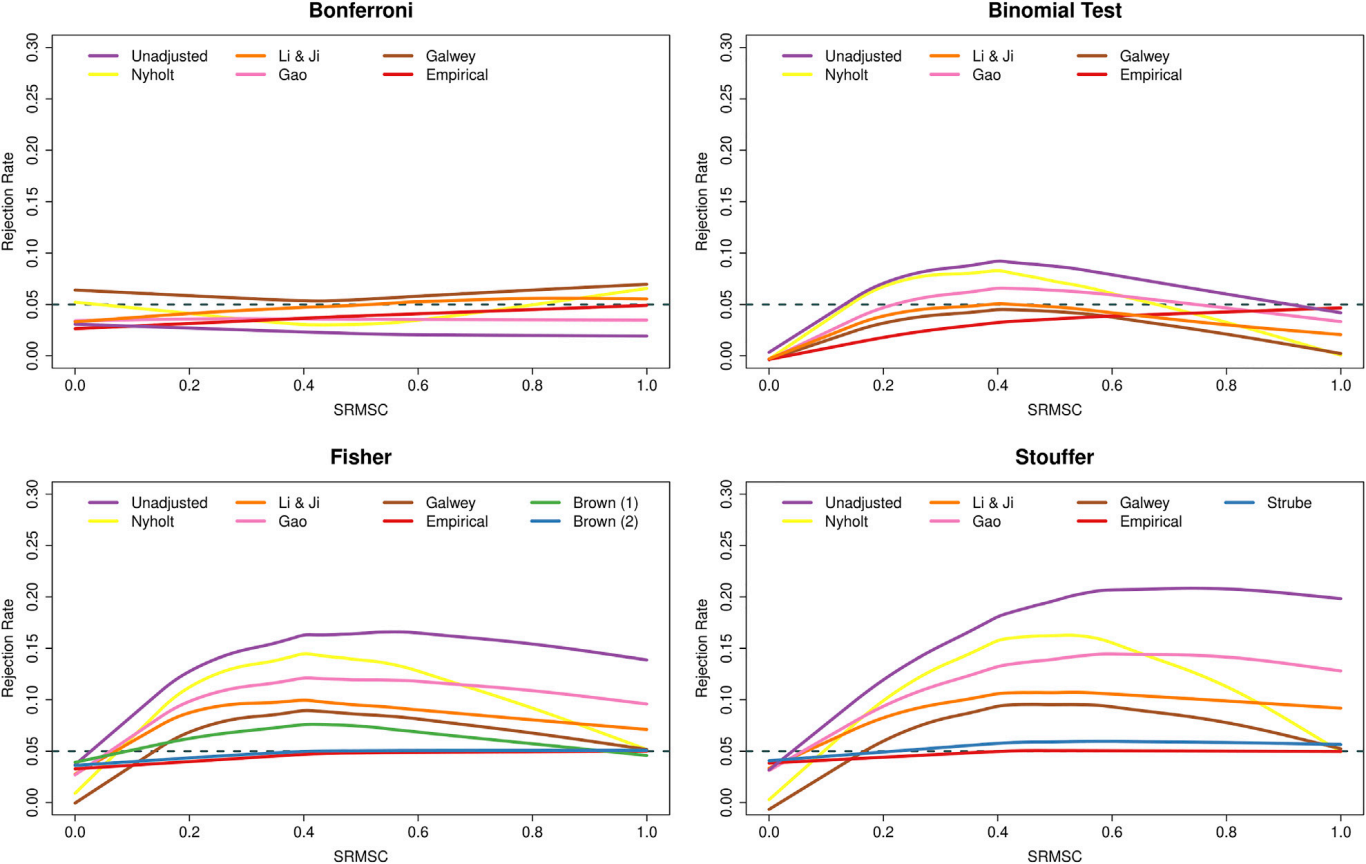
Type I error rates of methods for gene-based testing as a function of the square-root mean squared correlation (SRMSC). The horizontal grey dashed line corresponds to the nominal rejection rate of *α* = 0.05. Brown (1) and Brown (2) refer to the one- and two-sided versions of Brown’s method (i.e., Eqs 13,14, respectively).

Figure 3 shows the type I error rates as a function of the SRMSC values. As expected, the figure points out the increasingly conservative behavior of the Bonferroni method as the SNPs within the genes become more dependent, while Fisher’s and Stouffer’s methods then become liberal. The conservative behavior of the binomial test under independence is also expected (due to the discrete nature of the binomial distribution, the type I error rate of the test will not exceed *α* = 0.05, but will often fall well below it). More surprising is the fact that the type I error rate of the method was essentially nominal for genes with very strong LD. To understand this phenomenon, consider a gene with *k* SNPs in perfect LD. In that case, all (two-sided) *p*-values are identical and hence either none or all *k* SNPs are significant. Since the latter will happen (under the joint null) with probability *α*, the test will exhibit nominal performance under this extreme scenario. With respect to the adjustments, the various PCA-based approaches again had the effect of counteracting the conservativeness of the Bonferroni method, while leading to a reduction in the type I error rates of the other methods. For all methods, adjusting with the use of empirical distributions was most successful when LD is strong, while Brown’s method, although slightly conservative under independence, and performed well over the range of SRMSC values. Strube’s method performed similarly, but with some slight inflation for larger SRMSC values.

Supplementary Figures S4, S5 show the type I error rates of the methods as a function of the average correlation and the SD of the correlations of the SNPs within the genes. Here, we again find that changes in these characteristics have essentially no impact on the performance of Brown’s method, as well as when using empirical distributions in combination with Fisher’s and Strube’s methods. Interestingly, the one-sided version of Brown’s method performed similarly to the two-sided one when the average LD was larger than ≈0.3 .

Supplementary Figures S6, S7 display the type I error rates of the methods as a function of the average MAFs and their SD within the genes. The performance of Brown’s method and Fisher’s method with the empirical distribution adjustment was again not affected substantially by these factors except that these methods were slightly conservative when the average MAF was below 0.1 within the genes. Stouffer’s method adjusted based on empirical distributions and its generalization to dependence was also relatively robust to both factors.

#### 3.2.2 Statistical Power

**FIGURE 4:**
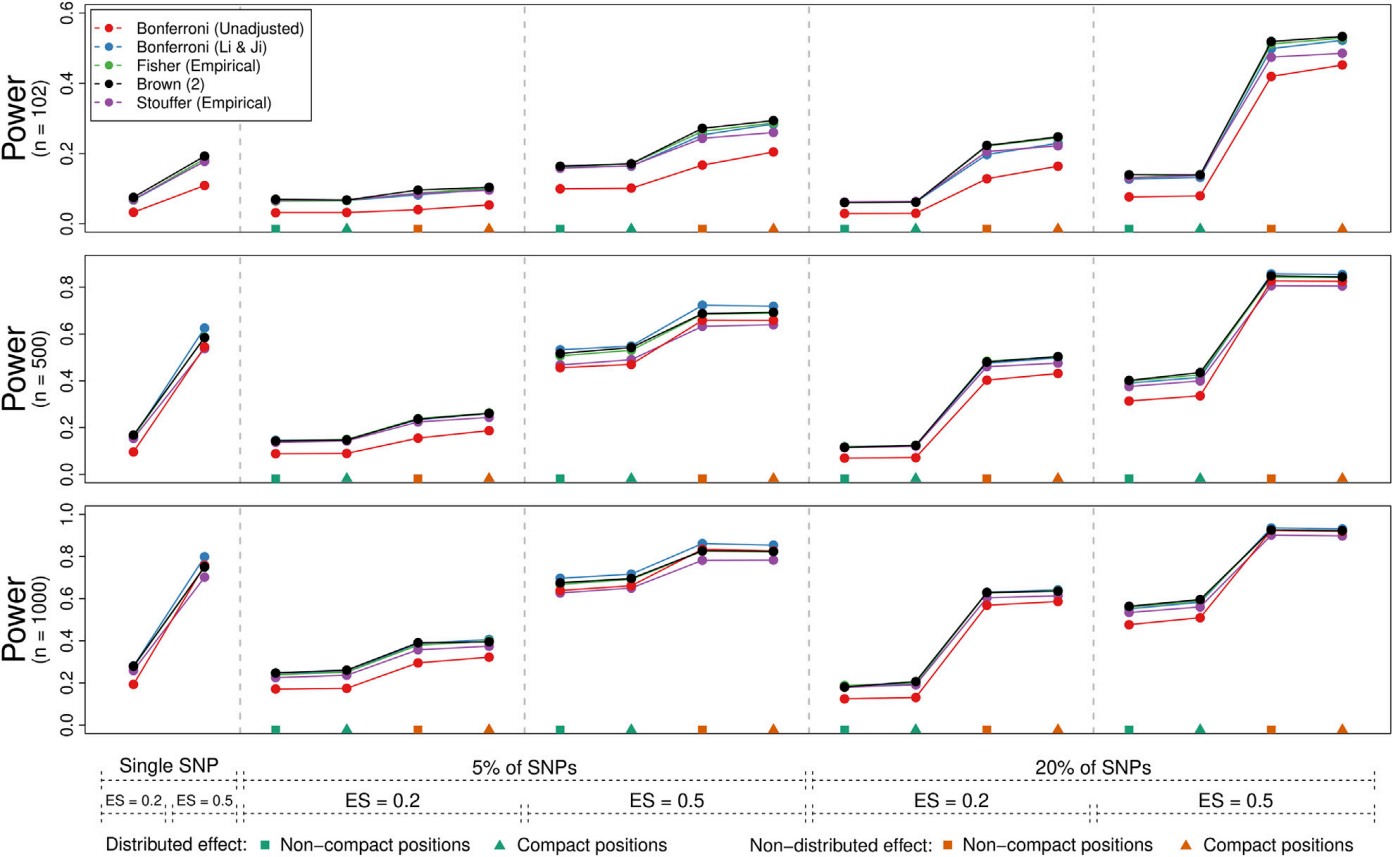
Power comparison of five methods for gene-based testing for three different sample sizes (*n* = 102, 500, and 1000). The points represent the average rejection rate of a method under a specific scenario as indicated by the labels/symbols under the *x*-axis (number/percent of SNPs that are associated with the phenotype, the effect size, whether the effect was distributed over the significant SNPs, and whether significant SNPs were compactly positioned within the genes). Brown (2) refers to the two-sided version of Brown’s method (i.e., Eq. 13).

Figure 4 illustrates the power of the methods (averaged over genes) for the 54 conditions where the joint null hypothesis was false. Each panel corresponds to one of three sample size conditions (i.e., 102, 500, and 1,000), while the *x*-axis indicates the condition, starting with the two “single SNP” conditions (with effect sizes of 0.2 vs. 0.5) followed by the 16 “multiple SNPs” conditions (with either 5% or 20% of SNPs selected, an effect sizes of 0.2 or 0.5, a non-distributed or distributed effect, and either non-compact or compact SNP positions). We only show the power rates for the (unadjusted) Bonferroni method and those method and adjustment combinations that could control the type I error rate on average (i.e., the Bonferroni method with the Li & Ji adjustment, Brown’s method, and use of empirical distributions in combination with Fisher’s and Stouffer’s methods).

As expected, power increased with the sample size, with the effect size, when a higher percentage of SNPs was associated with the phenotype, and when the effect was not distributed across these SNPs. Whether the selected SNPs fell into a compact region of the gene or were distributed throughout had comparatively little influence on the results. The (unadjusted) Bonferroni method typically had lower power compared to the other methods especially for lower sample sizes. Also, Stouffer’s method adjusted with the use of empirical distributions tended to have slightly lower power compared to the other adjusted methods. While differences between the other methods were often negligible, a slight power advantage could be observed for the Bonferroni method adjusted with the Li & Ji correction when a single SNP or a low percentage of them contained a strong signal. Otherwise, Brown’s method tended to have a slight power advantage.

To compare the computational efficiency of the methods, Supplementary Figure S8 presents the average computation times (based on 500 iterations) of the unadjusted methods along with the Li & Ji, empirical (using 10^4^ samples), and dependence adjustments (the latter only for the Fisher and Stouffer methods) for a set of genes with {10, 25, 50, 100, 199, 254, 492, 897, and 1150} SNPs. While the unadjusted methods show no noteworthy increase in computational times as a function of the gene size, the results show that the use of the adjustments does come at the cost of increased computational times, less so for the Li & Ji adjustment and more so when using the Brown and Strube methods. Finally, as expected, the empirical methods demand the highest amount of computer time (although this can be mitigated to some extent; see Cinar and Viechtbauer (2022) for details). The computation times for the different test types, however, did not appear to vary substantially.

## 4 Discussion

In this paper, we described some common methods for gene-based testing that combine the *p*-values of individual SNPs within genes (or that are clustered within some other higher-level functional structure) to test the joint null hypothesis that none of the SNPs within a gene are associated with the phenotype of interest. Along the way, we described a variety of adjustment techniques to incorporate LD information into this process. To examine and compare the type I error rates and power of the methods, we conducted an extensive simulation study based on HapMap data. While the (unadjusted) Bonferroni method guarantees that the type I error rate is never larger than the chosen significance level for all genes, the results show that this comes at the cost of a decrease in power for detecting genes that contain SNPs associated with the phenotype of interest.

Other methods for gene-based testing require adjustments based on the LD structure to ensure that their type I error rate is close to the nominal level on average. Doing so can increase the power for detecting “significant genes”, but this in turn can lead to an inflated type I error rate for some of the individual genes. We would consider this an acceptable risk under two conditions. First, the variability in the rates for individual genes should be low (to avoid excessively inflated type I error rates for particular genes). Moreover, the method should provide adequate control of the type I error rate regardless of the characteristics of the genes.

Among the various methods examined, the extension of Brown’s method to two-sided tests comes closest to fulfilling these requirements. It had a nominal type I error rate on average and the lowest variability in the rates for individual genes (also when compared against the unadjusted Bonferroni method). The highest type I error rate observed across all 30,910 genes was 0.091, but this value might reflect at least in part simulation error, as the Bonferroni method also had inflated rates for 470 (1.5%) of the genes, with a maximum rate equal to 0.069. To further examine this, we repeated the simulation for these 470 genes using 10^6^ iterations (see Supplementary Figure S10 for boxplots of the type I error rates of the Bonferroni and Brown’s method). Now, only 34 of these genes still had a type I error rate above 0.05 with the Bonferroni method, with a maximum rate of 0.056. In contrast, the highest type I error rate of Brown’s method was then 0.058, although (as expected) a higher number (188 out of these 470 genes) still had a rate above 0.05. Finally, the results based on all 30,910 genes showed that the Bonferroni method became increasingly conservative for genes with a larger number of SNPs or SNPs that were in stronger LD, while the performance of Brown’s method was essentially independent of the various gene characteristics examined (except for some slight conservativeness when the degree of LD was very weak).

Another consideration in this context is the relative performance of the methods depending on whether the ‘signal’ is concentrated in a single SNP or distributed over a larger number of them. The Bonferroni method–which focuses on the lowest *p*-value among the SNPs within a gene–might be at an advantage under the former scenario, while methods that can aggregate signals across multiple SNPs (such as Fisher’s and Stouffer’s method and versions thereof adjusted to account for dependence) would be expected to be more powerful in the latter case. However, under the conditions studied, the (unadjusted) Bonferroni method was never able to outperform Brown’s method even when only a single SNP was strongly associated with the phenotype. Only when combined with the adjustment by Li & Ji did the Bonferroni method show a slight power advantage under this scenario. Brown’s method may therefore be particularly advantageous when studying complex diseases where relatively small associations are likely to be spread across many SNPs and multiple genes (Neale and Sham, 2004; Moskvina et al., 2011).

We also considered how an estimate of the effective number of tests can be used to adjust other methods besides the Bonferroni or Tippett methods (to which such adjustments are typically applied). However, none of these generalizations yielded nominal type I error rates on average. On the other hand, combining the Bonferroni method with the estimate of Li and Ji (2005) did perform adequately and, as mentioned above, may be of interest when the signal is concentrated in a single SNP. Our findings are in line with those by Wen and Lu (2011) who showed that the method by Li and Ji (2005) performs better than other effective number of tests adjustments.

Finally, we explored methods that mimic “proper” permutation tests by using pseudo replicates of the *p*-values to construct the empirical distributions needed for such tests. This approach greatly reduces the computation time (and can even be used when the raw data are not available) and produces results that are quite similar to those of conventional permutation techniques (Lin, 2005; Conneely and Boehnke, 2007; Liu et al., 2010; Moskvina et al., 2011). However, the results of our simulation study show that the performance of this approach depends on the method used for combining the *p*-values. Moreover, the type I error rate of these pseudo permutation tests either tended to be slightly conservative or, when the type I error rate was nominal on average, they offered no power advantage over Brown’s method.

There are, however, a few issues that require further discussion. First, as mentioned at the beginning of Section 2, the methods discussed in the present manuscript assume that the *p*-values follow a Uniform (0, 1) distribution under the null hypothesis. In our simulation study, the association between the SNPs and case-control status was tested using the Cochran-Armitage trend test (as is often done in practice when assuming an additive model). When using the typical normal approximation for conducting this test, the *p*-values only follow a uniform distribution asymptotically. While an exact version of this test is also available (Williams, 1988), the discrete nature of the test (since it is based on the frequency counts in a contingency table) can still make the exact *p*-values slightly conservative under the null hypothesis. In general though, the uniform assumption should hold when the sample size underlying the *p*-values is sufficiently large. Moreover, as this is a common issue for all of the methods described, it should not affect the relative performance of the methods.

Furthermore, in this paper, we focused on methods for combining *p*-values that can explicitly incorporate information from the LD matrix into their computation. Other recently proposed techniques for combining *p*-values, such as the Cauchy combination test (Liu et al., 2019; Liu and Xie, 2020) and the harmonic mean *p*-value method (Wilson, 2019), do not make use of LD information, but still provide control of the type I error rate under dependence. Now that the present results indicate the most advantageous methods that directly make use of the LD matrix, a further step will be a comparison of these method with those that do not.

Similarly, gene-based testing can also be conducted using modeling techniques (see Chapman and Whittaker, 2008; Ionita-Laza et al., 2013; Moskvina et al., 2012, for examples); however, such techniques require access to the raw genotype data. The focus of the present paper was on methods that avoid this requirement, but the relative performances of model-based methods and methods for combining *p*-values is an important subject to be examined in the future.

Finally, in our simulation, we focused on the gene regions in the HapMap data. As is well-known, SNPs in intergenic regions may play an important role in gene regulation and therefore may also be associated with a phenotype of interest (Ionita-Laza et al., 2013). Methods for combining *p*-values can also be utilized for synthesizing information from such genome regions as long as the *p*-values and LD matrices are derived accordingly. One could, for example, treat such regions as separate sets, or include intergenic SNPs with their neighboring genes.

In conclusion, the present results indicate that the two-sided version of Brown’s method is a potentially attractive alternative to the use of the Bonferroni correction and other methods for gene-based testing. It is generally able to control the type I error rate and can lead to increased power, especially when associations are spread across multiple SNPs and genes. Those are the circumstances characterized by complex diseases where shifting the focus to higher functional structures may in fact be particularly advantageous.

## Data Availability

The original contributions presented in the study are included in the article/Supplementary Material, further inquiries can be directed to the corresponding author.
