# Supplementary material for: A Comparison of Methods for Gene-Based Testing That Account for Linkage Disequilibrium
Source: Front Genet. 2022 May 5;13:867724. doi: 10.3389/fgene.2022.867724 (PMC9117705; doi:10.3389/fgene.2022.867724)
Supplement: Supplementary file 1 [file DataSheet1.PDF]

A comparison of methods for gene-based testing that account for linkage disequilibrium

Supplementary Materials

Ozan Cinar
Wolfgang Viechtbauer
Maastricht University

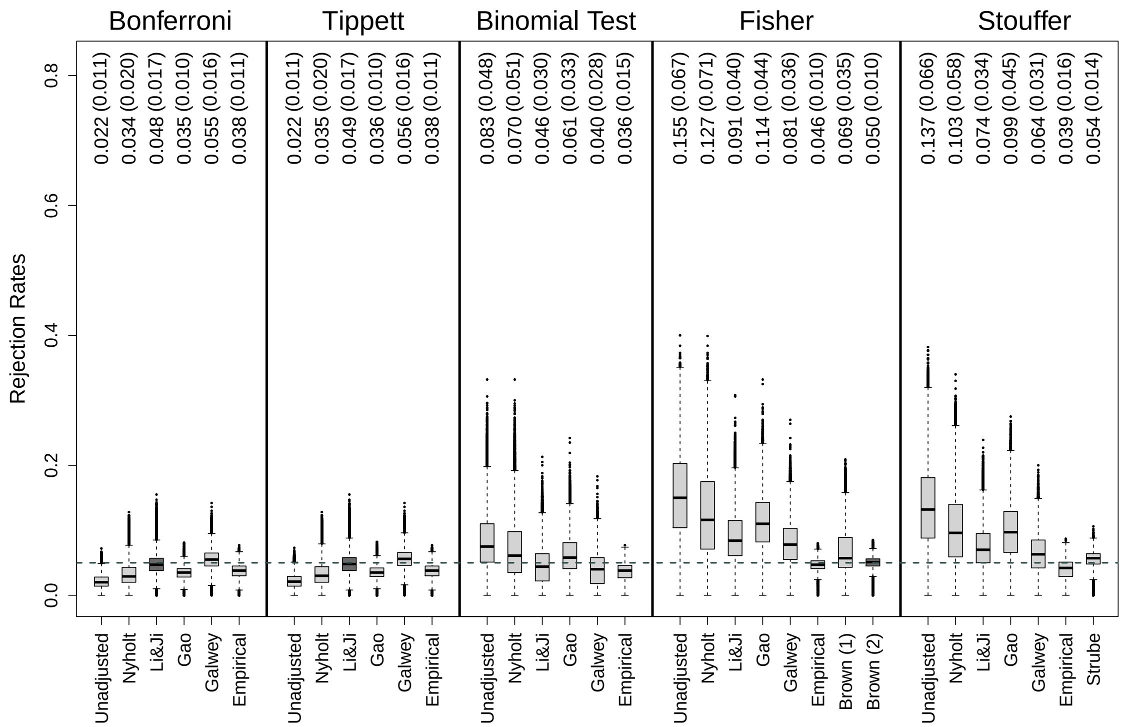

Figure S1: Type I error rates of methods for gene-based testing when applied to the bootstrap samples of size 102. The numbers above the boxplots show the mean (SD) rejection rates of the methods. The horizontal grey dashed line corresponds to the nominal rejection rate of $\alpha = 0.05$.

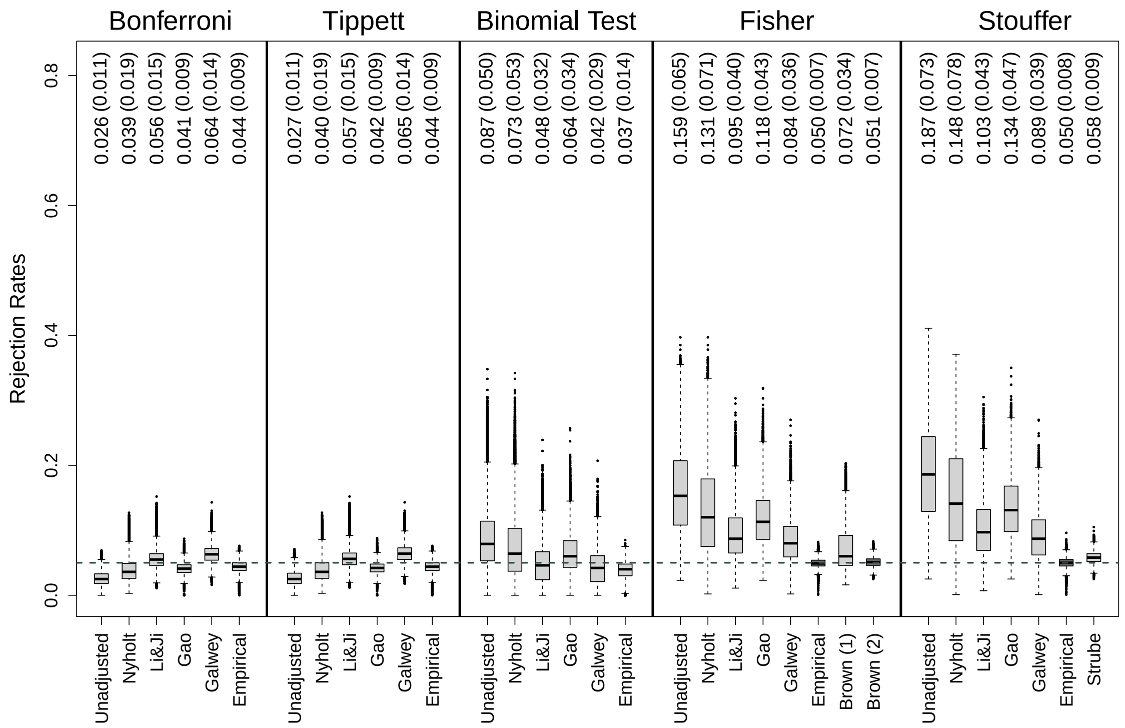

Figure S2: Type I error rates of methods for gene-based testing when applied to the bootstrap samples of size 500. The numbers above the boxplots show the mean (SD) rejection rates of the methods. The horizontal grey dashed line corresponds to the nominal rejection rate of $\alpha = 0.05$.

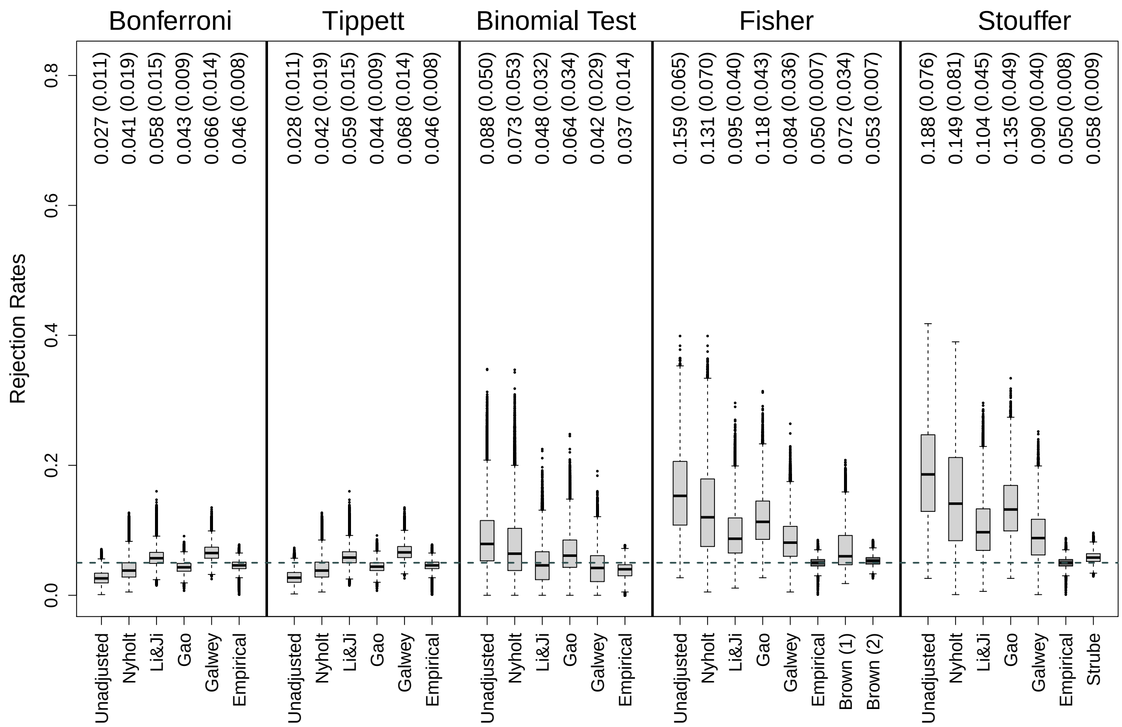

Figure S3: Type I error rates of methods for gene-based testing when applied to the bootstrap samples of size 1000. The numbers above the boxplots show the mean (SD) rejection rates of the methods. The horizontal grey dashed line corresponds to the nominal rejection rate of $\alpha = 0.05$.

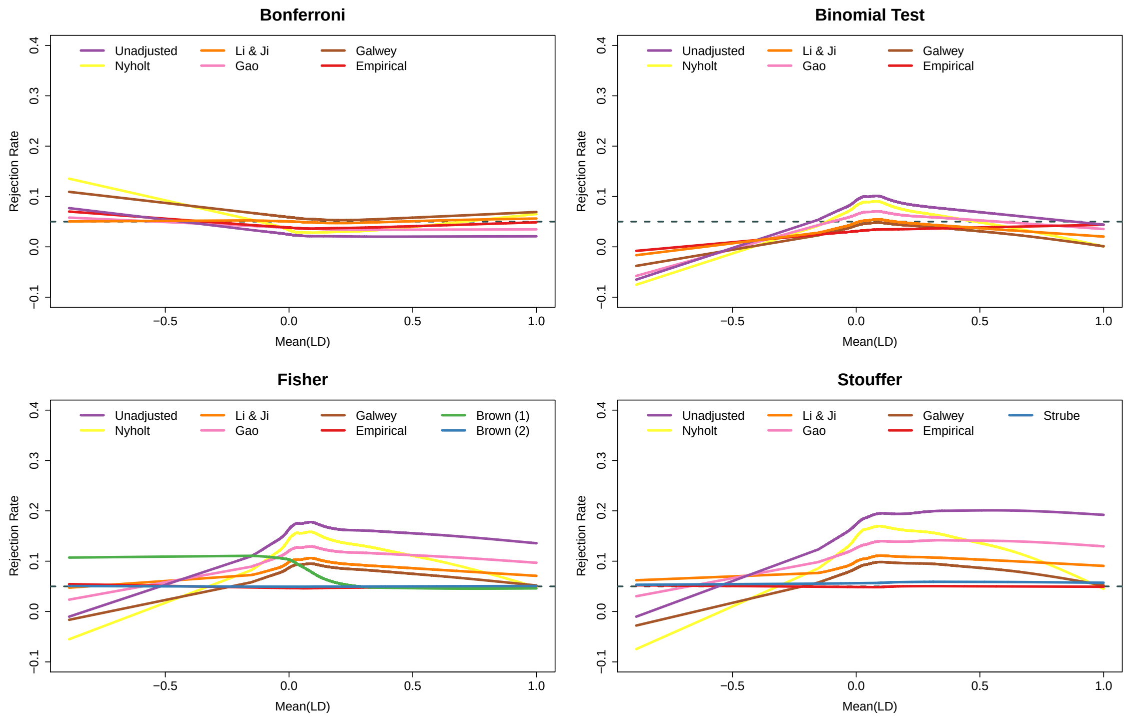

Figure S4: Type I error rates of methods for gene-based testing as a function of the average correlation of the SNPs. The horizontal grey dashed line corresponds to the nominal rejection rate of $\alpha = 0.05$.

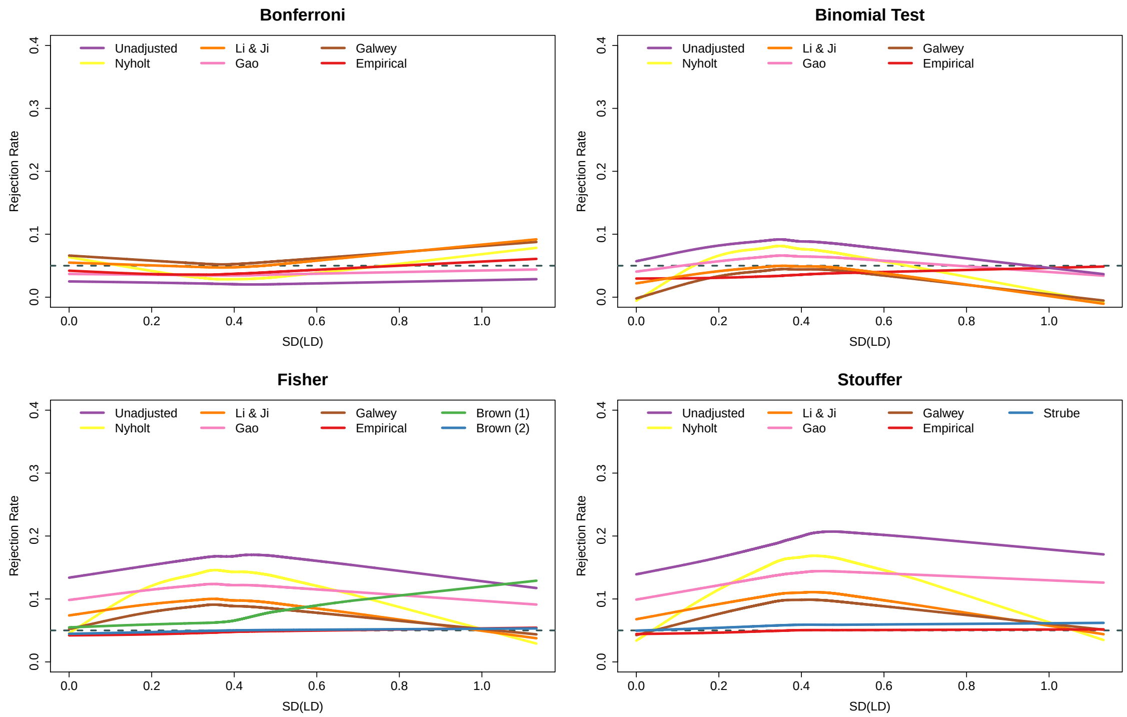

Figure S5: Type I error rates of methods for gene-based testing as a function of the standard deviation of the correlations of the SNPs. The horizontal grey dashed line corresponds to the nominal rejection rate of $\alpha = 0.05$.

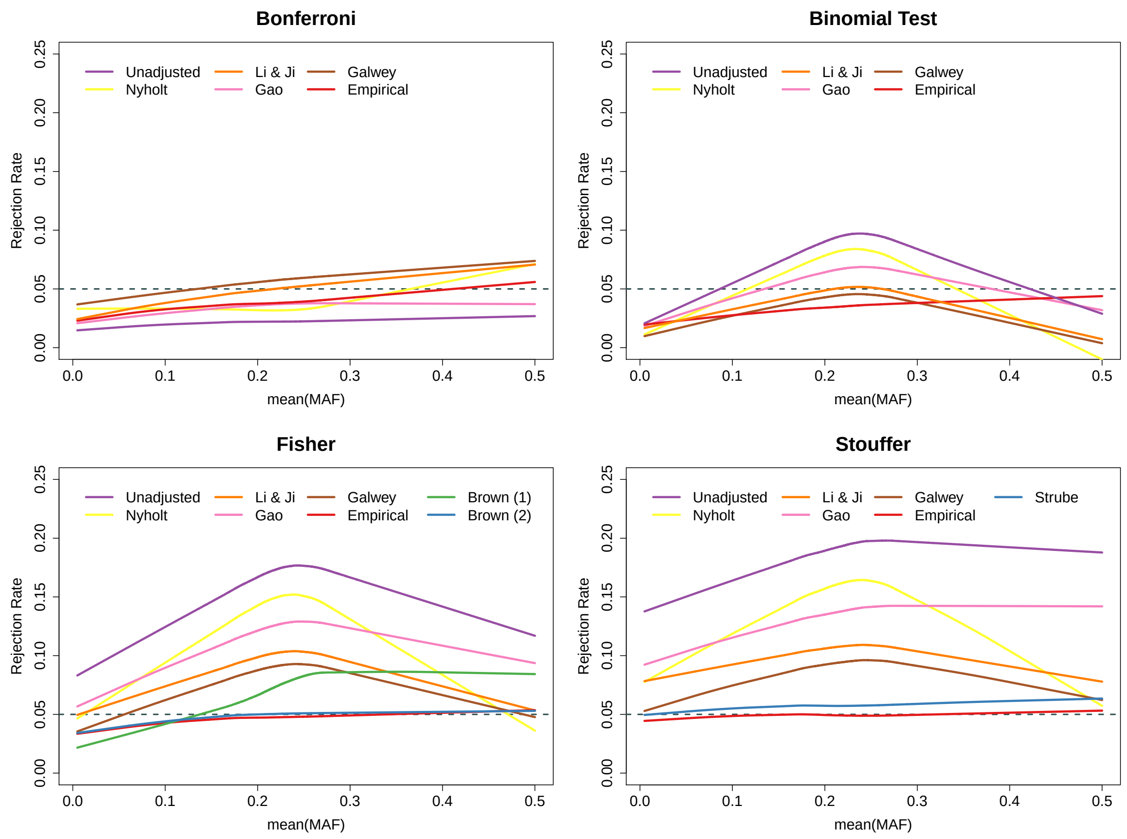

Figure S6: Type I error rates of methods for gene-based testing as a function of the average MAF statistics of the SNPs. The horizontal grey dashed line corresponds to the nominal rejection rate of $\alpha = 0.05$.

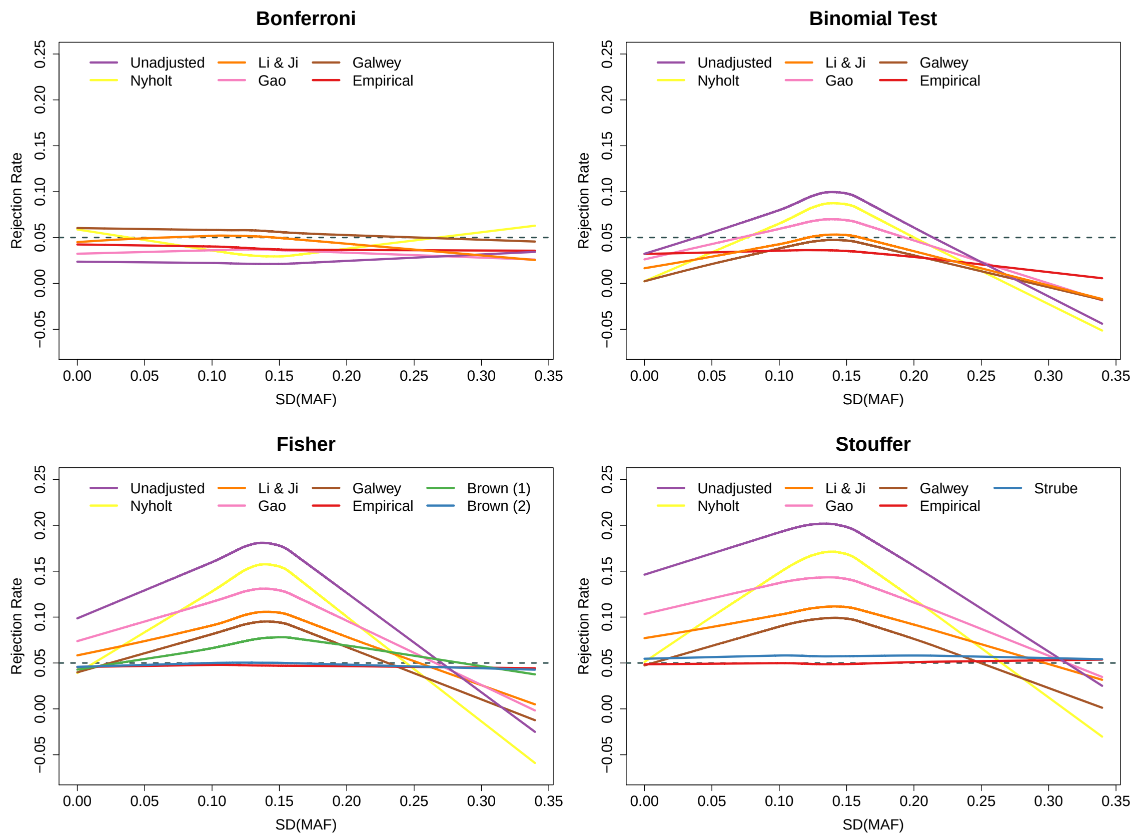

Figure S7: Type I error rates of methods for gene-based testing as a function of the standard deviation of the MAF statistics of the SNPs. The horizontal grey dashed line corresponds to the nominal rejection rate of $\alpha = 0.05$.

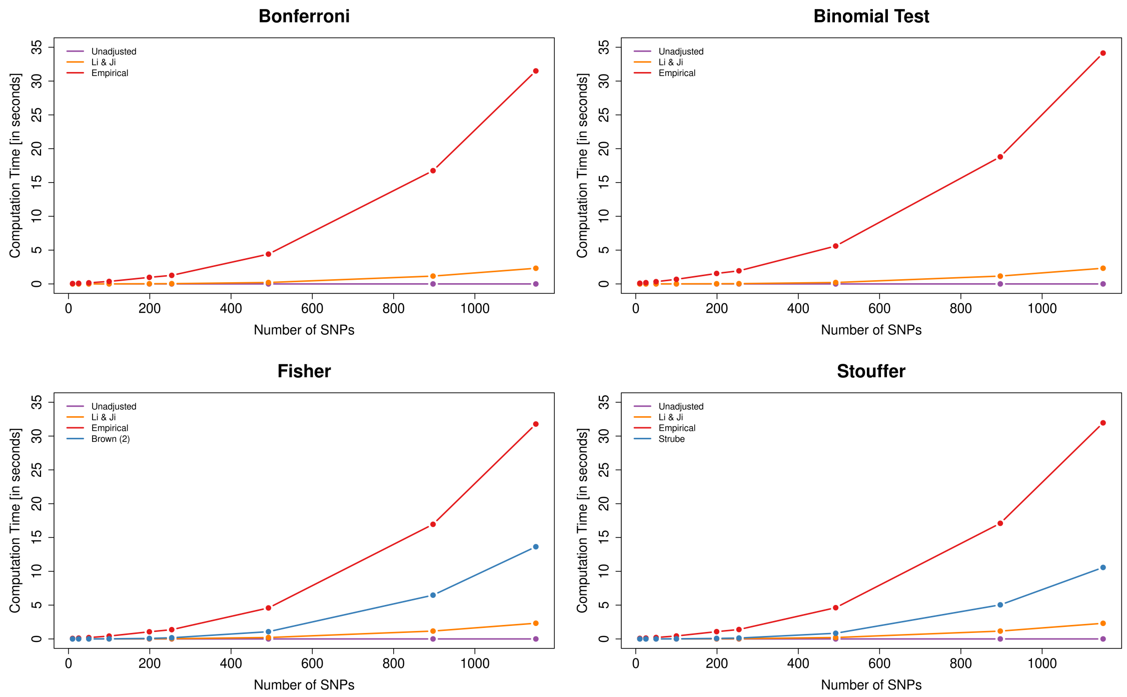

Figure S8: Average computation times (based on 500 iterations) of the unadjusted Bonferroni method, binomial test, Fisher's method, and Stouffer's method along with the Li & Ji, empirical (using 10^4 samples), and dependence adjustments (the latter only for the Fisher and Stouffer methods). The various adjustments are presented in different colors. Brown (2) refers to the two-sided version of Brown's method. The x-axis corresponds to the number of SNPs in the genes $\{10,$ 25, 50, 100, 199, 254, 492, 897, $1150\}$, whereas the y-axis present the computation time of deriving the combined p-value using a Intel i7-7700HQ CPU.

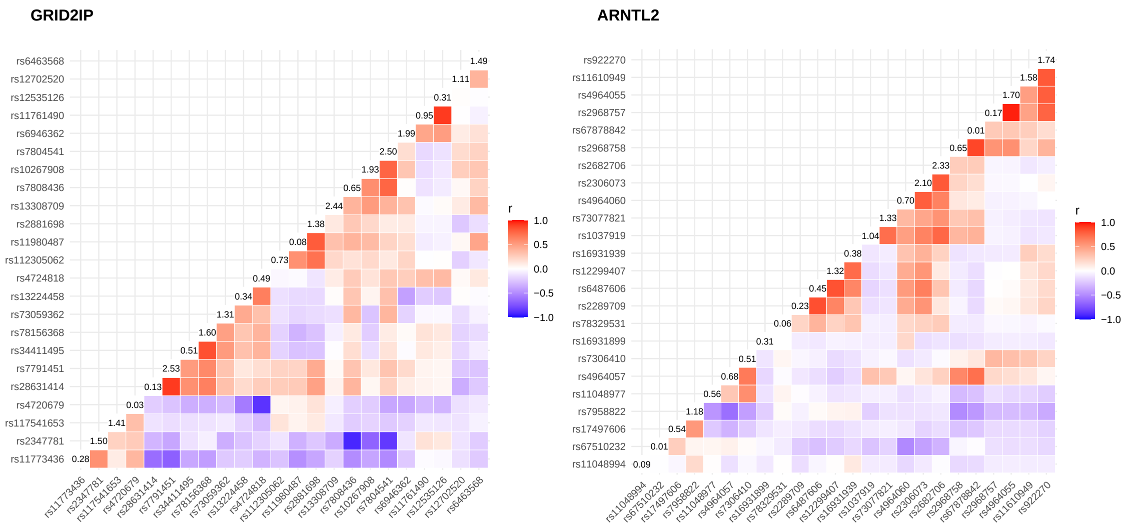

Figure S9: LD maps (allelic correlations) for the *GRID2IP* and *ARNTL2* genes. The values along the diagonals of the heatmaps show the individual $-\log_{10}(p)$-transformed values of the SNPs.

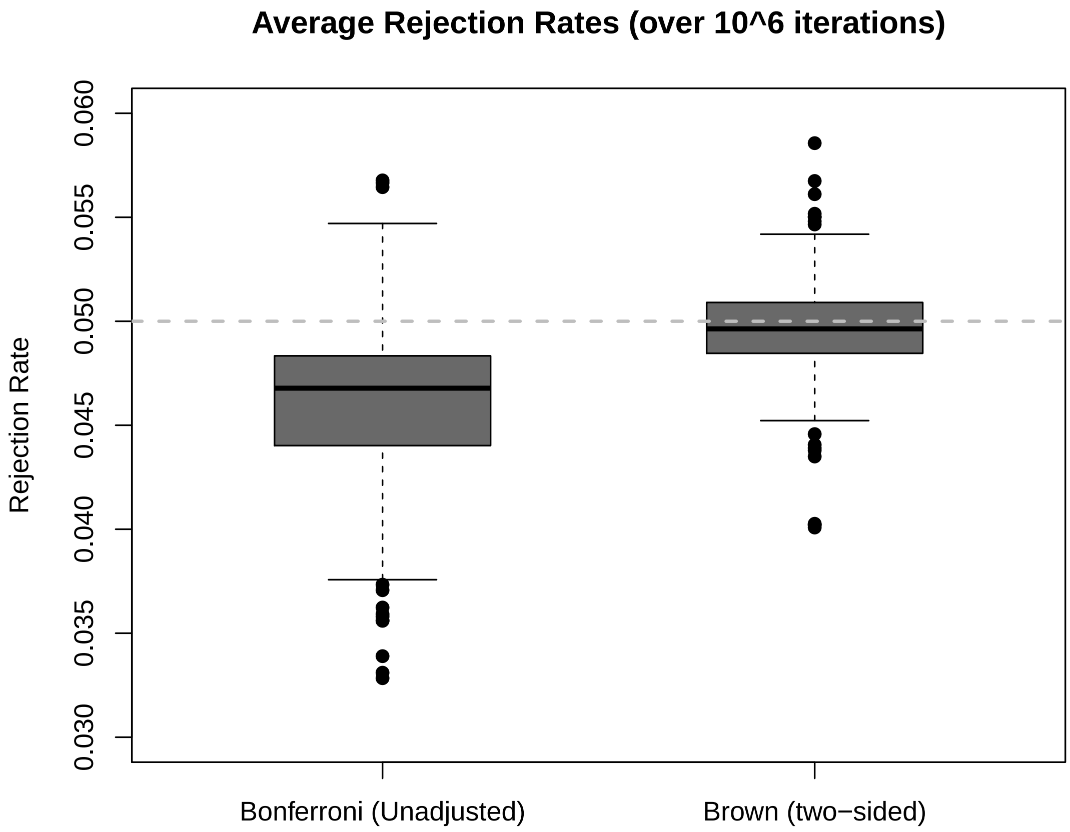

Figure S10: Type I error rates of the Bonferroni and Brown's method applied to 470 genes that had an inflated Type I error rate with the Bonferroni method under the original simulation (with 1,000 iterations) when repeating the simulation using 1,000,000 iterations.

S11. Production of the illustrative example in R

```
# installing and loading the poolr package
# remotes::install_github("ozancinar/poolr")
library(poolr)
```

```
pval.grid2ip <- c(0.523867, 0.031784, 0.039298, 0.923166, 0.749155, 0.002977,
                  0.310855, 0.024953, 0.048951, 0.454377, 0.325193, 0.18527,
                  0.825963, 0.041549, 0.003599, 0.011868, 0.00314, 0.225177,
                  0.010242, 0.111822, 0.494852, 0.077975, 0.032064)
pval.arntl2 <- c(0.813566, 0.966331, 0.291202, 0.065472, 0.277919, 0.210565,
                 0.309, 0.494661, 0.868732, 0.587439, 0.352955, 0.047845,
                 0.421138, 0.091675, 0.046717, 0.200075, 0.008009, 0.004662,
                 0.222106, 0.984071, 0.674515, 0.019844, 0.026476, 0.018167)

LD.grid2ip <- structure(c(1, -0.25, -0.2289, -0.139, 0.4655, -0.2263, -0.1023,
                          -0.1525, -0.0757, 0.3887, 0.2324, -0.2195, -0.0546,
                          0.3603, 0.2305, 0.2913, 0.155, -0.0566, -0.0994,
                          -0.0095, -0.0173, -0.0589, 0.1177, -0.25, 1, 0.9113,
                          0.4353, 0.2228, -0.6886, 0.2292, 0.6019, 0.5136,
                          -0.2446, 0.306, -0.3813, 0.3086, 0.0338, 0.3068,
                          0.1228, 0.1939, -0.1427, -0.2554, 0.0452, 0.1224,
                          0.057, 0.1733, -0.2289, 0.9113, 1, 0.4845, 0.274,
                          -0.6304, 0.2631, 0.6414, 0.5673, -0.3499, 0.3832,
                          -0.3154, 0.3244, 0.066, 0.2359, 0.0442, 0.0952,
                          -0.1197, -0.2126, 0.0459, 0.1892, 0.0607, 0.259,
                          -0.139, 0.4353, 0.4845, 1, 0.7967, -0.3836, 0.705,
                          -0.2587, -0.2538, -0.2357, 0.2853, -0.2399, -0.1549,
                          0.1134, 0.1012, 0.2018, 0.1037, 0.1098, 0.1478,
                          -0.0481, -0.1616, -0.0453, -0.1118, 0.4655, 0.2228,
                          0.274, 0.7967, 1, -0.4812, 0.5635, -0.3247, -0.2605,
                          0.0374, 0.3911, -0.3366, -0.1743, 0.3207, 0.2262,
                          0.3535, 0.1721, 0.0627, 0.0678, -0.0598, -0.1593,
                          -0.0812, -0.0224, -0.2263, -0.6886, -0.6304, -0.3836,
                          -0.4812, 1, -0.2573, -0.4205, -0.3788, -0.1307,
                          -0.4986, 0.5653, -0.2288, -0.1923, -0.5108, -0.3867,
                          -0.2307, 0.0994, 0.3746, -0.0275, -0.2131, -0.0156,
                          -0.3239, -0.1023, 0.2292, 0.2631, 0.705, 0.5635,
                          -0.2573, 1, -0.1905, -0.1877, -0.199, 0.1497,
                          -0.1129, -0.1287, 0.2029, 0.1148, 0.1985, 0.2196,
                          0.1436, 0.0521, 0.0078, -0.1332, -0.0045,
                          -0.0327, -0.1525, 0.6019, 0.6414, -0.2587, -0.3247,
                          -0.4205, -0.1905, 1, 0.8397, -0.1885, 0.1128,
                          -0.0663, 0.4892, -0.0679, 0.1008, -0.2128, 0.0257,
                          -0.1177, -0.3435, 0.1206, 0.2987, 0.1489, 0.3874,
                          -0.0757, 0.5136, 0.5673, -0.2538, -0.2605, -0.3788,
                          -0.1877, 0.8397, 1, -0.17, 0.1734, -0.1268, 0.5134,
                          -0.0097, 0.146, -0.1459, -0.0164, -0.132, -0.3458,
                          0.0846, 0.3227, 0.1174, 0.3892, 0.3887, -0.2446,
```

```
-0.3499, -0.2357, 0.0374, -0.1307, -0.199, -0.1885,
-0.17, 1, 0.0351, -0.1191, -0.1449, 0.1054, 0.1841,
0.2554, 0.0979, -0.1191, -0.1325, 0.0122, 0.0096,
-0.0026, 0.0309, 0.2324, 0.306, 0.3832, 0.2853,
0.3911, -0.4986, 0.1497, 0.1128, 0.1734, 0.0351,
1, -0.9179, 0.3683, 0.3881, 0.7577, 0.5756, 0.0096,
-0.2185, -0.2033, -0.0863, 0.2965, -0.1214, 0.2703,
-0.2195, -0.3813, -0.3154, -0.2399, -0.3366, 0.5653,
-0.1129, -0.0663, -0.1268, -0.1191, -0.9179, 1,
-0.346, -0.359, -0.8506, -0.6744, -0.1104, 0.2357,
0.2703, 0.1213, -0.2552, 0.1537, -0.1917, -0.0546,
0.3086, 0.3244, -0.1549, -0.1743, -0.2288, -0.1287,
0.4892, 0.5134, -0.1449, 0.3683, -0.346, 1, -0.1359,
0.381, -0.2542, -0.1883, -0.0888, -0.2915, -0.0316,
0.4381, -0.0324, 0.3237, 0.3603, 0.0338, 0.066,
0.1134, 0.3207, -0.1923, 0.2029, -0.0679, -0.0097,
0.1054, 0.3881, -0.359, -0.1359, 1, 0.3957, 0.514,
0.3133, -0.0975, -0.0671, 0.0214, 0.0146, -0.0216,
0.0978, 0.2305, 0.3068, 0.2359, 0.1012, 0.2262,
-0.5108, 0.1148, 0.1008, 0.146, 0.1841, 0.7577,
-0.8506, 0.381, 0.3957, 1, 0.7585, 0.1755, -0.2293,
-0.3812, -0.1244, 0.3263, -0.1597, 0.289, 0.2913,
0.1228, 0.0442, 0.2018, 0.3535, -0.3867, 0.1985,
-0.2128, -0.1459, 0.2554, 0.5756, -0.6744, -0.2542,
0.514, 0.7585, 1, 0.2976, -0.1855, -0.2155, -0.1002,
0.0572, -0.1433, 0.1456, 0.155, 0.1939, 0.0952,
0.1037, 0.1721, -0.2307, 0.2196, 0.0257, -0.0164,
0.0979, 0.0096, -0.1104, -0.1883, 0.3133, 0.1755,
0.2976, 1, -0.195, -0.3769, 0.5028, -0.4097, 0.4621,
0.2583, -0.0566, -0.1427, -0.1197, 0.1098, 0.0627,
0.0994, 0.1436, -0.1177, -0.132, -0.1191, -0.2185,
0.2357, -0.0888, -0.0975, -0.2293, -0.1855, -0.195,
1, 0.3316, -0.1068, -0.1912, -0.0982, -0.2911,
-0.0994, -0.2554, -0.2126, 0.1478, 0.0678, 0.3746,
0.0521, -0.3435, -0.3458, -0.1325, -0.2033, 0.2703,
-0.2915, -0.0671, -0.3812, -0.2155, -0.3769, 0.3316,
1, -0.3234, -0.5716, -0.2973, -0.8709, -0.0095,
0.0452, 0.0459, -0.0481, -0.0598, -0.0275, 0.0078,
0.1206, 0.0846, 0.0122, -0.0863, 0.1213, -0.0316,
0.0214, -0.1244, -0.1002, 0.5028, -0.1068, -0.3234,
1, -0.2358, 0.9098, 0.3576, -0.0173, 0.1224, 0.1892,
-0.1616, -0.1593, -0.2131, -0.1332, 0.2987, 0.3227,
0.0096, 0.2965, -0.2552, 0.4381, 0.0146, 0.3263,
0.0572, -0.4097, -0.1912, -0.5716, -0.2358, 1,
-0.2167, 0.647, -0.0589, 0.057, 0.0607, -0.0453,
-0.0812, -0.0156, -0.0045, 0.1489, 0.1174, -0.0026,
-0.1214, 0.1537, -0.0324, -0.0216, -0.1597, -0.1433,
0.4621, -0.0982, -0.2973, 0.9098, -0.2167, 1, 0.3331,
0.1177, 0.1733, 0.259, -0.1118, -0.0224, -0.3239,
-0.0327, 0.3874, 0.3892, 0.0309, 0.2703, -0.1917,
0.3237, 0.0978, 0.289, 0.1456, 0.2583, -0.2911,
-0.8709, 0.3576, 0.647, 0.3331, 1), .Dim = c(23L,
                                             23L))
```

```
LD.arntl2 <- structure(c(1, 0.2549, -0.1571, -0.173, 0.4807, -0.0798, -0.2967,
                         0.4976, 0.2142, 0.8081, 0.1294, 0.2998, -0.0792,
                         -0.0489, -0.0437, -0.1388, 0.1152, 0.2572, 0.1385,
                         -0.1012, -0.1131, -0.0041, -0.101, 0.1318, 0.2549, 1,
                         -0.2397, -0.2639, 0.2772, -0.1221, -0.4448, 0.2895,
                         0.8735, 0.1765, 0.7152, 0.1242, -0.1528, -0.0893,
                         0.0991, -0.0107, -0.166, -0.0973, -0.141, 0.3286,
                         0.4006, 0.1745, 0.2684, -0.1553, -0.1571, -0.2397, 1,
                         -0.2324, -0.1575, -0.1113, 0.5271, -0.1525, -0.2619,
                         -0.1935, -0.3301, -0.2203, -0.0398, -0.0701, -0.0964,
                         0.2536, -0.1434, -0.1232, -0.1572, -0.0662, -0.0884,
                         -0.1853, -0.1445, -0.0985, -0.173, -0.2639, -0.2324,
                         1, -0.1317, -0.1229, -0.4548, -0.1679, -0.2954,
                         -0.2133, 0.2906, 0.5752, 0.0091, 0.0552, -0.1324,
                         0.0483, -0.0543, -0.1028, -0.1187, -0.0667, -0.0685,
                         -0.0941, -0.0554, -0.0108, 0.4807, 0.2772, -0.1575,
                         -0.1317, 1, -0.08, -0.2975, 0.9672, 0.5494, 0.7629,
                         0.1996, 0.3675, -0.0796, -0.034, -0.0597, -0.1298,
                         0.0053, -0.0883, -0.0073, -0.0595, -0.051, -0.0328,
                         -0.0435, 0.0254, -0.0798, -0.1221, -0.1113, -0.1229,
                         -0.08, 1, -0.211, -0.0774, -0.1368, -0.0985, -0.1684,
                         -0.1123, -0.0989, -0.002, 0.2026, -0.0369, -0.0966,
                         -0.0623, -0.0558, -0.0715, -0.0775, -0.1403, -0.1155,
                         -0.0805, -0.2967, -0.4448, 0.5271, -0.4548, -0.2975,
                         -0.211, 1, -0.288, -0.5029, -0.3653, -0.6223, -0.4159,
                         0.1925, 0.0221, -0.1291, 0.0545, 0.0273, 0.0661,
                         0.0586, -0.1228, -0.2027, -0.1184, -0.1021, -0.0607,
                         0.4976, 0.2895, -0.1525, -0.1679, 0.9672, -0.0774,
                         -0.288, 1, 0.5692, 0.7883, 0.1742, 0.3336, -0.0745,
                         -0.0358, -0.0548, -0.1425, 0.0197, -0.0854, 0.0023,
                         -0.0769, -0.0586, -0.0294, -0.0453, 0.0414, 0.2142,
                         0.8735, -0.2619, -0.2954, 0.5494, -0.1368, -0.5029,
                         0.5692, 1, 0.391, 0.608, 0.0715, -0.1613, -0.0801,
                         0.1236, -0.0425, -0.0868, -0.1172, -0.0864, 0.2893,
                         0.3732, 0.2195, 0.2592, -0.0446, 0.8081, 0.1765,
                         -0.1935, -0.2133, 0.7629, -0.0985, -0.3653, 0.7883,
                         0.391, 1, 0.0453, 0.2247, -0.0789, -0.0209, -9e-04,
                         -0.1659, 0.1946, 0.1855, 0.2018, -0.1136, -0.0978,
                         0.051, -0.0728, 0.2212, 0.1294, 0.7152, -0.3301,
                         0.2906, 0.1996, -0.1684, -0.6223, 0.1742, 0.608,
                         0.0453, 1, 0.6682, -0.1314, -0.0165, 0.0473, 0.0721,
                         -0.1423, -0.1467, -0.2073, 0.2822, 0.3213, 0.1391,
                         0.242, -0.0998, 0.2998, 0.1242, -0.2203, 0.5752,
                         0.3675, -0.1123, -0.4159, 0.3336, 0.0715, 0.2247,
                         0.6682, 1, -0.0356, 0.0475, -0.1272, 0.0204, -0.0639,
                         -0.1243, -0.1521, -0.0316, -0.0588, -0.0905, -0.0215,
                         -0.0381, -0.0792, -0.1528, -0.0398, 0.0091, -0.0796,
                         -0.0989, 0.1925, -0.0745, -0.1613, -0.0789, -0.1314,
                         -0.0356, 1, 0.0048, -0.1656, -0.0051, -0.0422, 0.1172,
                         0.0083, -0.0834, -0.0833, -0.0913, -0.0146, -0.1431,
                         -0.0489, -0.0893, -0.0701, 0.0552, -0.034, -0.002,
                         0.0221, -0.0358, -0.0801, -0.0209, -0.0165, 0.0475,
```

```
                           0.0048, 1, 0.2017, -0.0864, 0.3894, 0.3039, 0.2288,
                           -0.072, -0.0653, 0.2352, 0.2672, 0.222, -0.0437,
                           0.0991, -0.0964, -0.1324, -0.0597, 0.2026, -0.1291,
                           -0.0548, 0.1236, -9e-04, 0.0473, -0.1272, -0.1656,
                           0.2017, 1, -0.5105, 0.5168, 0.3107, 0.4292, 0.3634,
                           0.4951, 0.7886, 0.6359, 0.4344, -0.1388, -0.0107,
                           0.2536, 0.0483, -0.1298, -0.0369, 0.0545, -0.1425,
                           -0.0425, -0.1659, 0.0721, 0.0204, -0.0051, -0.0864,
                           -0.5105, 1, -0.2672, -0.1639, -0.2233, -0.1877,
                           -0.2582, -0.4097, -0.3296, -0.221, 0.1152, -0.166,
                           -0.1434, -0.0543, 0.0053, -0.0966, 0.0273, 0.0197,
                           -0.0868, 0.1946, -0.1423, -0.0639, -0.0422, 0.3894,
                           0.5168, -0.2672, 1, 0.6141, 0.8348, -0.1297, -0.1784,
                           0.6492, 0.3035, 0.8442, 0.2572, -0.0973, -0.1232,
                           -0.1028, -0.0883, -0.0623, 0.0661, -0.0854, -0.1172,
                           0.1855, -0.1467, -0.1243, 0.1172, 0.3039, 0.3107,
                           -0.1639, 0.6141, 1, 0.7353, -0.079, -0.1048, 0.4007,
                           0.2379, 0.29, 0.1385, -0.141, -0.1572, -0.1187,
                           -0.0073, -0.0558, 0.0586, 0.0023, -0.0864, 0.2018,
                           -0.2073, -0.1521, 0.0083, 0.2288, 0.4292, -0.2233,
                           0.8348, 0.7353, 1, -0.1081, -0.1488, 0.5412, 0.1084,
                           0.6212, -0.1012, 0.3286, -0.0662, -0.0667, -0.0595,
                           -0.0715, -0.1228, -0.0769, 0.2893, -0.1136, 0.2822,
                           -0.0316, -0.0834, -0.072, 0.3634, -0.1877, -0.1297,
                           -0.079, -0.1081, 1, 0.7225, 0.4584, 0.5542, -0.1105,
                           -0.1131, 0.4006, -0.0884, -0.0685, -0.051, -0.0775,
                           -0.2027, -0.0586, 0.3732, -0.0978, 0.3213, -0.0588,
                           -0.0833, -0.0653, 0.4951, -0.2582, -0.1784, -0.1048,
                           -0.1488, 0.7225, 1, 0.6301, 0.7515, -0.1344, -0.0041,
                           0.1745, -0.1853, -0.0941, -0.0328, -0.1403, -0.1184,
                           -0.0294, 0.2195, 0.051, 0.1391, -0.0905, -0.0913,
                           0.2352, 0.7886, -0.4097, 0.6492, 0.4007, 0.5412,
                           0.4584, 0.6301, 1, 0.8084, 0.5487, -0.101, 0.2684,
                           -0.1445, -0.0554, -0.0435, -0.1155, -0.1021, -0.0453,
                           0.2592, -0.0728, 0.242, -0.0215, -0.0146, 0.2672,
                           0.6359, -0.3296, 0.3035, 0.2379, 0.1084, 0.5542,
                           0.7515, 0.8084, 1, 0.128, 0.1318, -0.1553, -0.0985,
                           -0.0108, 0.0254, -0.0805, -0.0607, 0.0414, -0.0446,
                           0.2212, -0.0998, -0.0381, -0.1431, 0.222, 0.4344,
                           -0.221, 0.8442, 0.29, 0.6212, -0.1105, -0.1344,
                           0.5487, 0.128, 1), .Dim = c(24L, 24L))
```

```
pval.grid2ip[1:5]
```

```
## [1] 0.523867 0.031784 0.039298 0.923166 0.749155
```

```
pval.arntl2[1:5]
```

```
## [1] 0.813566 0.966331 0.291202 0.065472 0.277919
```

```
LD.grid2ip[1:5, 1:5]
```

```
##          [,1]    [,2]    [,3]    [,4]   [,5]
## [1,]  1.0000 -0.2500 -0.2289 -0.1390 0.4655
```

```
## [2,] -0.2500  1.0000  0.9113  0.4353 0.2228
## [3,] -0.2289  0.9113  1.0000  0.4845 0.2740
## [4,] -0.1390  0.4353  0.4845  1.0000 0.7967
## [5,]  0.4655  0.2228  0.2740  0.7967 1.0000
```

```
LD.arntl2[1:5, 1:5]
```

```
##         [,1]    [,2]    [,3]    [,4]    [,5]
## [1,]  1.0000  0.2549 -0.1571 -0.1730  0.4807
## [2,]  0.2549  1.0000 -0.2397 -0.2639  0.2772
## [3,] -0.1571 -0.2397  1.0000 -0.2324 -0.1575
## [4,] -0.1730 -0.2639 -0.2324  1.0000 -0.1317
## [5,]  0.4807  0.2772 -0.1575 -0.1317  1.0000
```

```
# setting the seed for empirical adjustments
set.seed(1234)

# combining p-values
p.dat  <- pval.grid2ip
ld.dat <- LD.grid2ip

res.grid2ip <-matrix(
  c(bonferroni(p.dat)$p,
    bonferroni(p.dat, adjust = "nyholt", R = ld.dat)$p,
    bonferroni(p.dat, adjust = "liji", R = ld.dat)$p,
    bonferroni(p.dat, adjust = "gao", R = ld.dat)$p,
    bonferroni(p.dat, adjust = "galwey", R = ld.dat)$p,
    bonferroni(p.dat, adjust = "empirical", R = ld.dat, size = 1000000)$p,
    NA,
    tippett(p.dat)$p,
    tippett(p.dat, adjust = "nyholt", R = ld.dat)$p,
    tippett(p.dat, adjust = "liji", R = ld.dat)$p,
    tippett(p.dat, adjust = "gao", R = ld.dat)$p,
    tippett(p.dat, adjust = "galwey", R = ld.dat)$p,
    tippett(p.dat, adjust = "empirical", R = ld.dat, size = 1000000)$p,
    NA,
    binomtest(p.dat)$p,
    binomtest(p.dat, adjust = "nyholt", R = ld.dat)$p,
    binomtest(p.dat, adjust = "liji", R = ld.dat)$p,
    binomtest(p.dat, adjust = "gao", R = ld.dat)$p,
    binomtest(p.dat, adjust = "galwey", R = ld.dat)$p,
    binomtest(p.dat, adjust = "empirical", R = ld.dat, size = 1000000)$p,
    NA,
    fisher(p.dat)$p,
    fisher(p.dat, adjust = "nyholt", R = ld.dat)$p,
    fisher(p.dat, adjust = "liji", R = ld.dat)$p,
    fisher(p.dat, adjust = "gao", R = ld.dat)$p,
    fisher(p.dat, adjust = "galwey", R = ld.dat)$p,
    fisher(p.dat, adjust = "empirical", R = ld.dat, size = 1000000)$p,
    fisher(p.dat, adjust = "generalized", R = mvnconv(ld.dat, target = "m2lp"))$p,
    stouffer(p.dat)$p,
    stouffer(p.dat, adjust = "nyholt", R = ld.dat)$p,
    stouffer(p.dat, adjust = "liji", R = ld.dat)$p,
    stouffer(p.dat, adjust = "gao", R = ld.dat)$p,
    stouffer(p.dat, adjust = "galwey", R = ld.dat)$p,
```

```r
    stouffer(p.dat, adjust = "empirical", R = ld.dat, size = 1000000)$p,
    stouffer(p.dat, adjust = "generalized", R = mvnconv(ld.dat, target = "z"))$p),
    byrow = TRUE, nrow = 5)

p.dat  <- pval.arntl2
ld.dat <- LD.arntl2

res.arntl2 <-matrix(
  c(bonferroni(p.dat)$p,
    bonferroni(p.dat, adjust = "nyholt", R = ld.dat)$p,
    bonferroni(p.dat, adjust = "liji", R = ld.dat)$p,
    bonferroni(p.dat, adjust = "gao", R = ld.dat)$p,
    bonferroni(p.dat, adjust = "galwey", R = ld.dat)$p,
    bonferroni(p.dat, adjust = "empirical", R = ld.dat, size = 1000000)$p,
    NA,
    tippett(p.dat)$p,
    tippett(p.dat, adjust = "nyholt", R = ld.dat)$p,
    tippett(p.dat, adjust = "liji", R = ld.dat)$p,
    tippett(p.dat, adjust = "gao", R = ld.dat)$p,
    tippett(p.dat, adjust = "galwey", R = ld.dat)$p,
    tippett(p.dat, adjust = "empirical", R = ld.dat, size = 1000000)$p,
    NA,
    binomtest(p.dat)$p,
    binomtest(p.dat, adjust = "nyholt", R = ld.dat)$p,
    binomtest(p.dat, adjust = "liji", R = ld.dat)$p,
    binomtest(p.dat, adjust = "gao", R = ld.dat)$p,
    binomtest(p.dat, adjust = "galwey", R = ld.dat)$p,
    binomtest(p.dat, adjust = "empirical", R = ld.dat, size = 1000000)$p,
    NA,
    fisher(p.dat)$p,
    fisher(p.dat, adjust = "nyholt", R = ld.dat)$p,
    fisher(p.dat, adjust = "liji", R = ld.dat)$p,
    fisher(p.dat, adjust = "gao", R = ld.dat)$p,
    fisher(p.dat, adjust = "galwey", R = ld.dat)$p,
    fisher(p.dat, adjust = "empirical", R = ld.dat, size = 1000000)$p,
    fisher(p.dat, adjust = "generalized", R = mvnconv(ld.dat, target = "m2lp"))$p,
    stouffer(p.dat)$p,
    stouffer(p.dat, adjust = "nyholt", R = ld.dat)$p,
    stouffer(p.dat, adjust = "liji", R = ld.dat)$p,
    stouffer(p.dat, adjust = "gao", R = ld.dat)$p,
    stouffer(p.dat, adjust = "galwey", R = ld.dat)$p,
    stouffer(p.dat, adjust = "empirical", R = ld.dat, size = 1000000)$p,
    stouffer(p.dat, adjust = "generalized", R = mvnconv(ld.dat, target = "z"))$p),
  byrow = TRUE, nrow = 5)

colnames(res.grid2ip) <- colnames(res.arntl2) <- c("Unadj", "ChNy", "LiJi", "Gao", "Gal",
                                                   "Emp", "Dep")

rownames(res.grid2ip) <- rownames(res.arntl2) <- c("Bonferroni", "Tippett", "Binomial",
                                                   "Fisher", "Stouffer")

meff.grid2ip <- c(meff(LD.grid2ip, method = "nyholt"), meff(LD.grid2ip, method = "liji"),
                  meff(LD.grid2ip, method = "gao"), meff(LD.grid2ip, method = "galwey"))
```

```r
meff.arntl2 <- c(meff(LD.arntl2, method = "nyholt"), meff(LD.arntl2, method = "liji"),
                 meff(LD.arntl2, method = "gao"), meff(LD.arntl2, method = "galwey"))

names(meff.grid2ip) <- names(meff.arntl2) <- c("ChNy", "LiJi", "Gao", "Gal")
```

```r
round(res.grid2ip, 3)
```

```
##            Unadj  ChNy  LiJi   Gao   Gal   Emp   Dep
## Bonferroni 0.068 0.060 0.045 0.054 0.039 0.052    NA
## Tippett    0.066 0.058 0.044 0.052 0.038 0.051    NA
## Binomial   0.000 0.000 0.000 0.000 0.000 0.000    NA
## Fisher     0.000 0.000 0.000 0.000 0.000 0.002 0.001
## Stouffer   0.000 0.000 0.000 0.000 0.000 0.002 0.000
```

```r
round(res.arntl2, 3)
```

```
##            Unadj  ChNy  LiJi   Gao   Gal   Emp   Dep
## Bonferroni 0.112 0.103 0.065 0.084 0.065 0.082    NA
## Tippett    0.106 0.098 0.063 0.081 0.063 0.082    NA
## Binomial   0.000 0.001 0.004 0.002 0.004 0.011    NA
## Fisher     0.000 0.000 0.003 0.001 0.003 0.020 0.016
## Stouffer   0.001 0.001 0.009 0.003 0.009 0.030 0.023
```

```r
meff.grid2ip
```

```
## ChNy LiJi  Gao  Gal
##   20   15   18   13
```

```r
meff.arntl2
```

```
## ChNy LiJi  Gao  Gal
##   22   14   18   14
```
